# Supplementary figures and images for: Epigenomic characterization of latent HIV infection identifies latency regulating transcription factors
Source: PLoS Pathog. 2021 Feb 26;17(2):e1009346. doi: 10.1371/journal.ppat.1009346 (PMC7946360; doi:10.1371/journal.ppat.1009346)

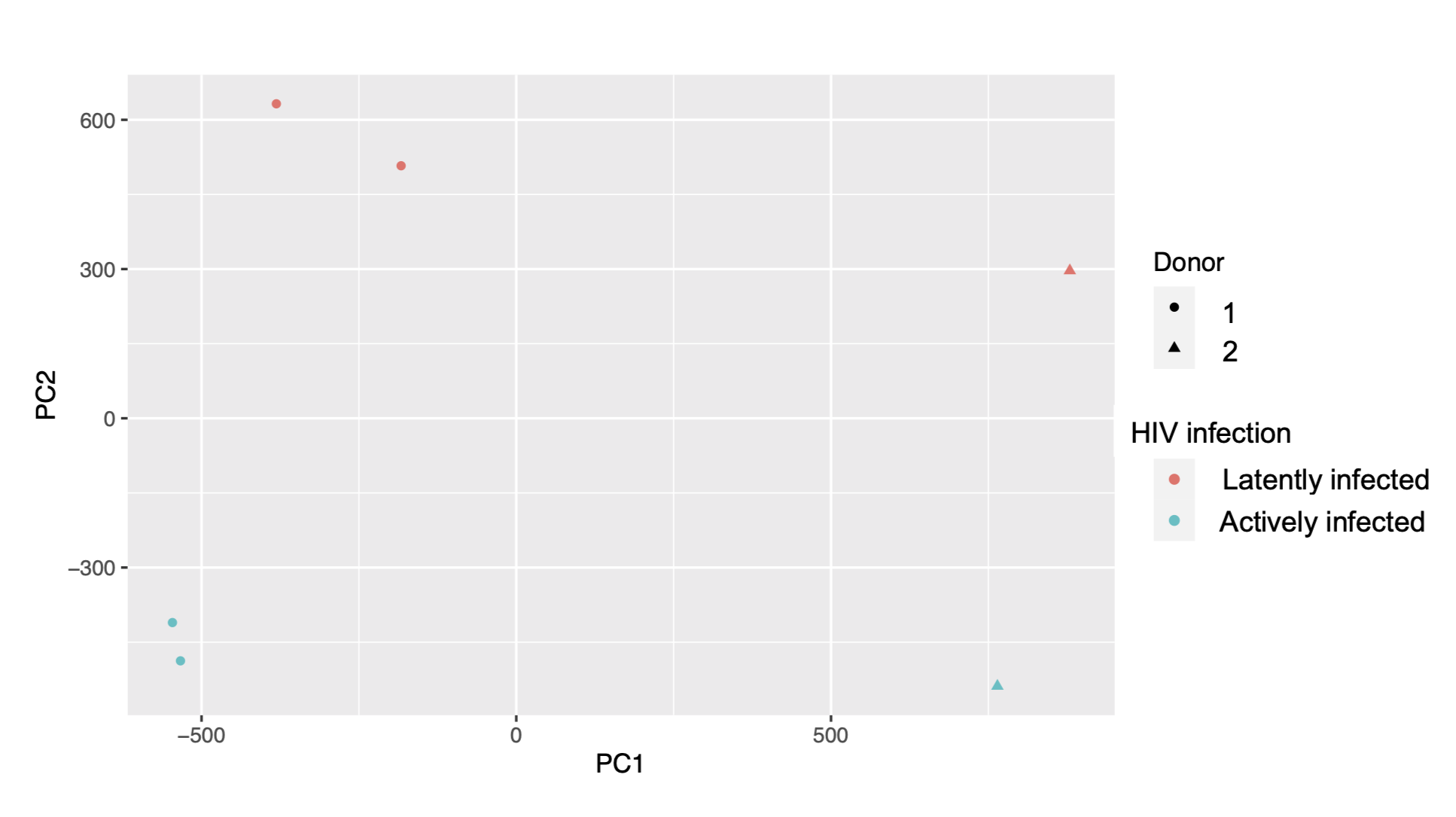

Supplement: S1 Fig — Principal component analysis was performed on ATACseq data from actively infected (GFP+, blue datapoints) and latently infected (GFP-, red datapoints) CD4 T cells. For Donor 1 (circles), two independent replicate experiments are shown. Donor 2 data represented by triangles. (TIF) [file ppat.1009346.s001.tif]

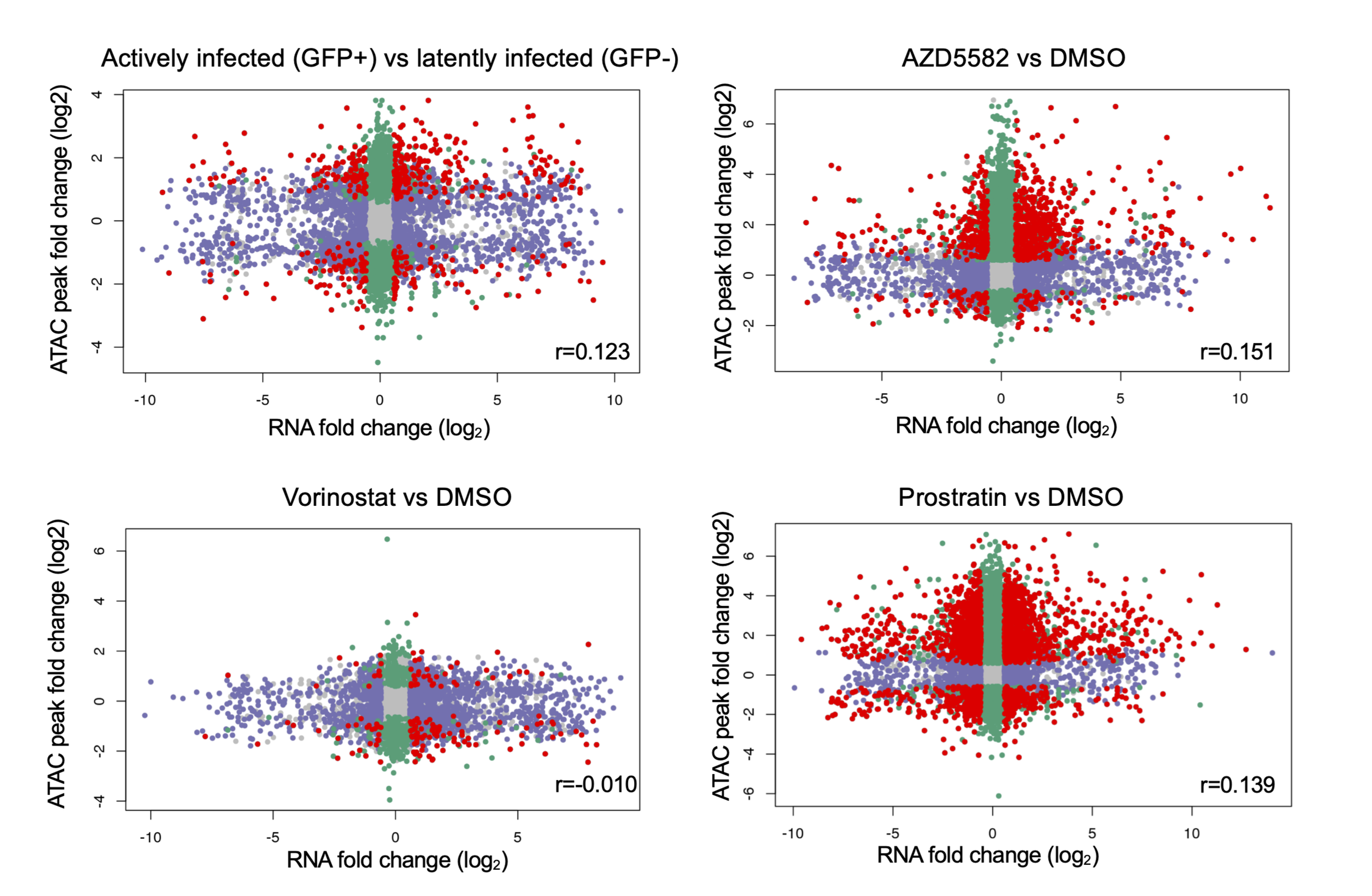

Supplement: S2 Fig — Fold changes for both RNA expression and ATACseq accessibility were calculated for each cellular gene and plotted. Each datapoint represents an individual gene. Data points are color coded by statistical significance–purple = genes with significant change to RNA transcript only (P<0.1, fold change >1.5), green = genes with significant change to ATACseq peak only (P<0.1, fold change >1.5), red = genes with significant change to both RNA transcript and ATACseq peak. Correlation coefficients for “red” genes between ATACseq peak change and RNA transcript change are indicated in the lower right corner of each panel. (TIF) [file ppat.1009346.s002.tif]

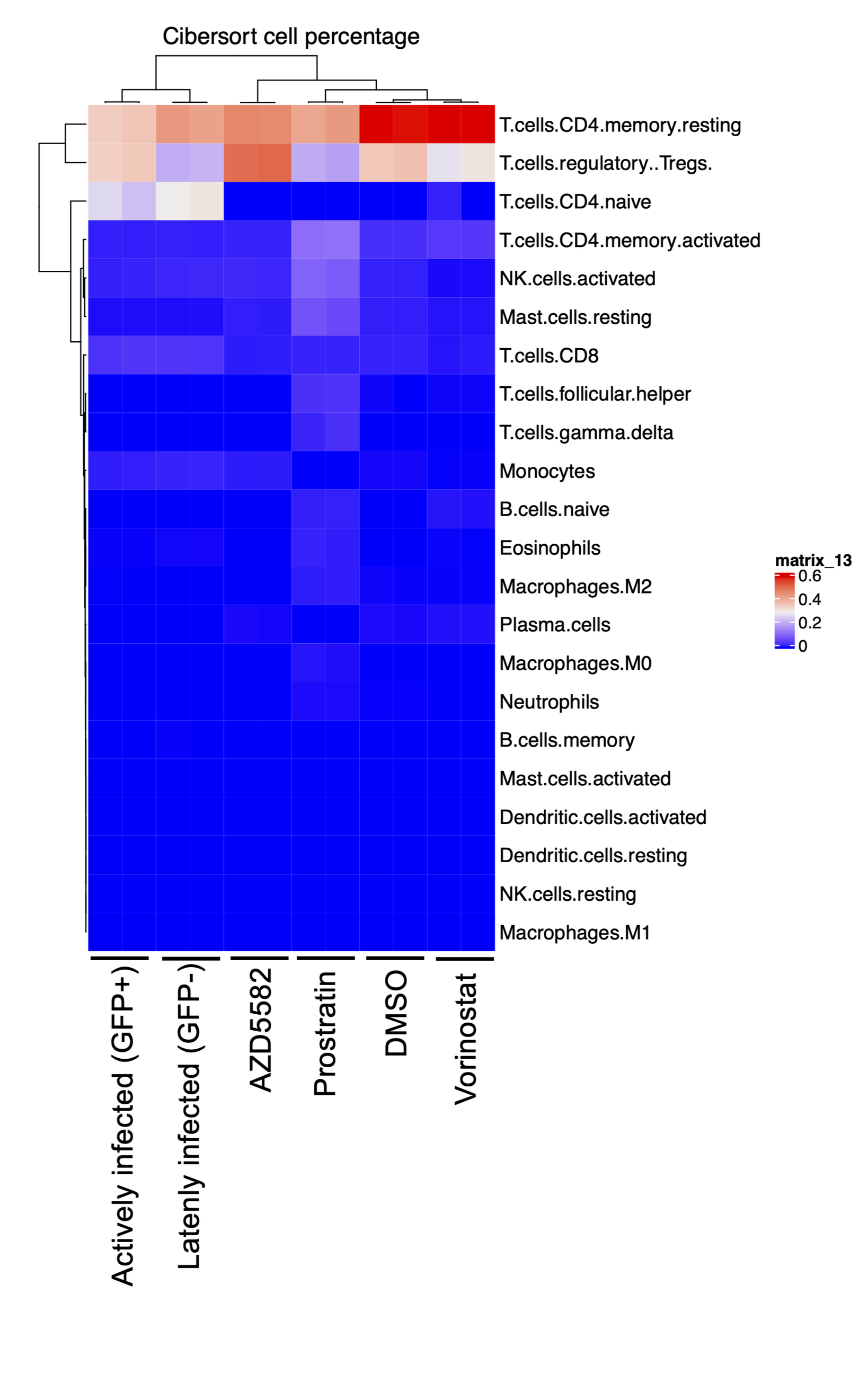

Supplement: S3 Fig — HIV latency model cells were profiled by RNAseq and compared to reference transcriptomes using the CIBERSORT machine learning method [35]. Enrichment of each experimental sample with hallmark signatures from immune cell reference profiles were calculated and plotted as a heatmap. (TIF) [file ppat.1009346.s003.tif]

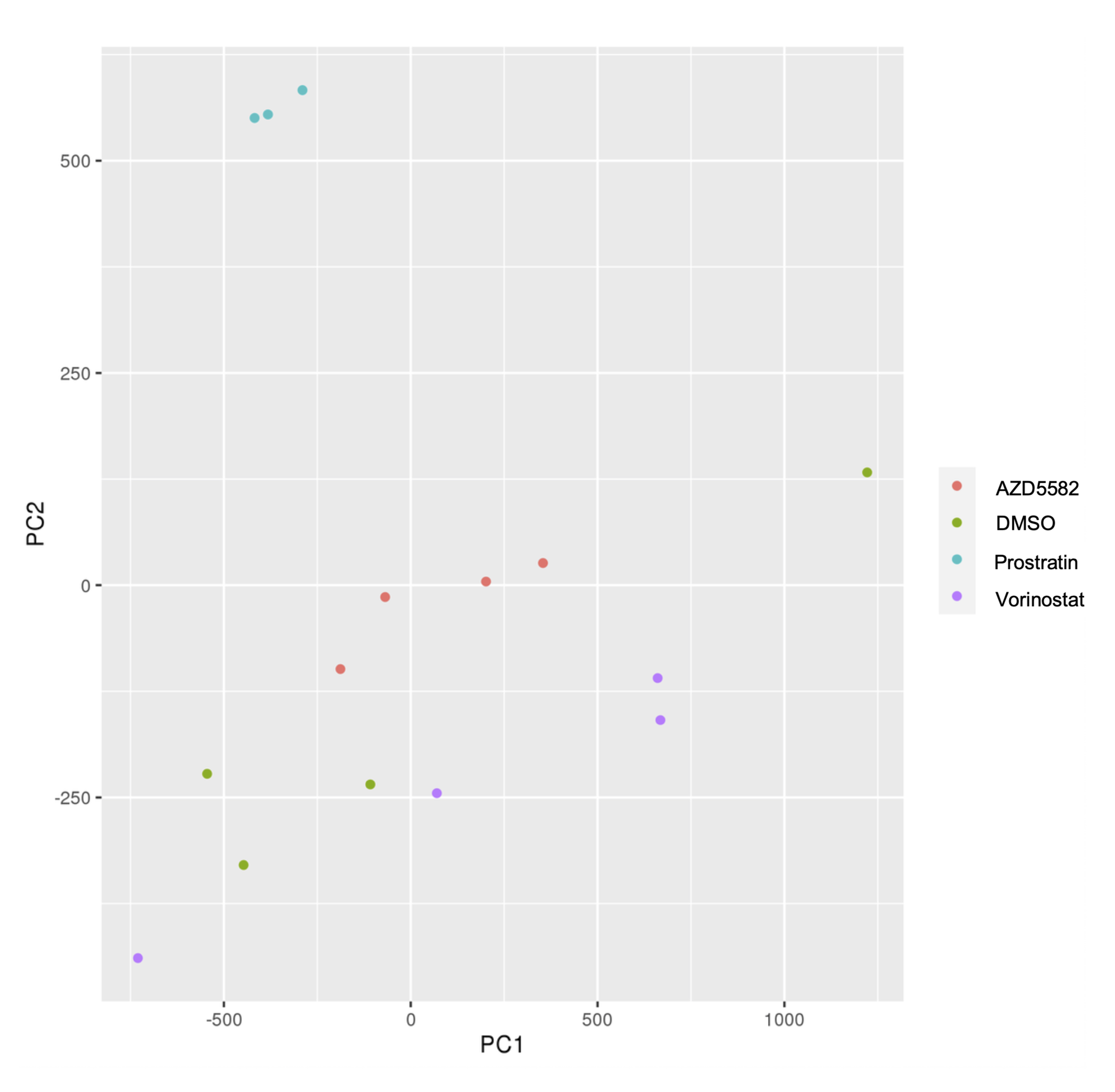

Supplement: S4 Fig — Principal component analysis was performed on ATACseq data from HIV-infected cells after 24h stimulation with three different LRAs or vehicle (DMSO). For all conditions, four independent replicate experiments are shown except for prostratin, for which three replicates were used. For all LRAs 250nM concentration was used. (TIF) [file ppat.1009346.s004.tif]

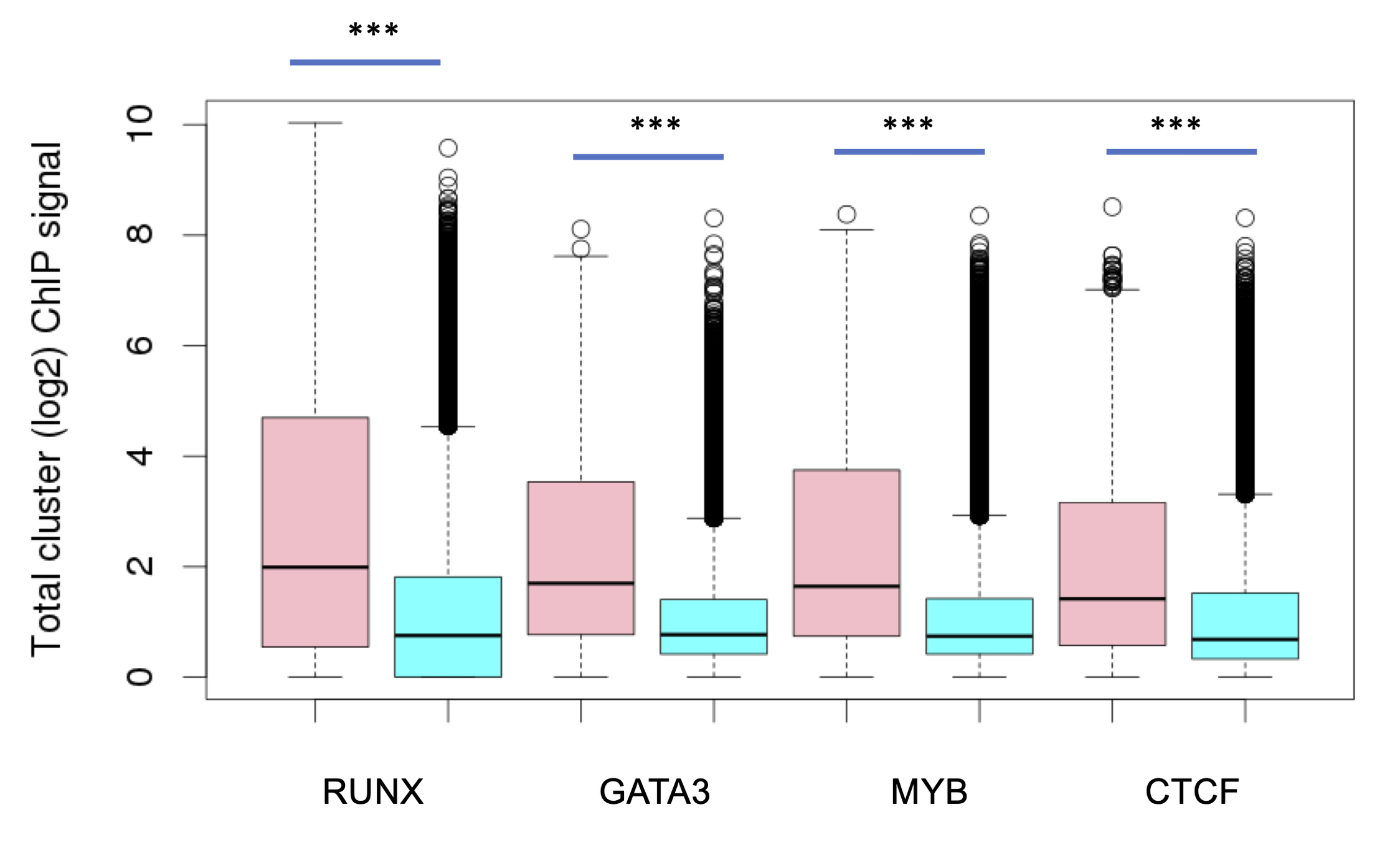

Supplement: S5 Fig — Differentially accessible DNA regions between actively infected (GFP+) cells and latently infected (GFP-) cells were compared to ChIPseq datasets for RUNX1, GATA3, MYB, and CTCF [35]. For each accessible peak, a ChIPseq signal score was calculated for the TFs indicated. Box plots of the summed peak cluster ChIPseq scores are shown for peaks that exhibited significantly increased accessibility in GFP+ cells (pink) versus those that showed no significant change (blue). P values for differences between the samples was calculated by Welch two sample t-test (S8 Table; *** denotes P<0.0001). (TIF) [file ppat.1009346.s005.tif]

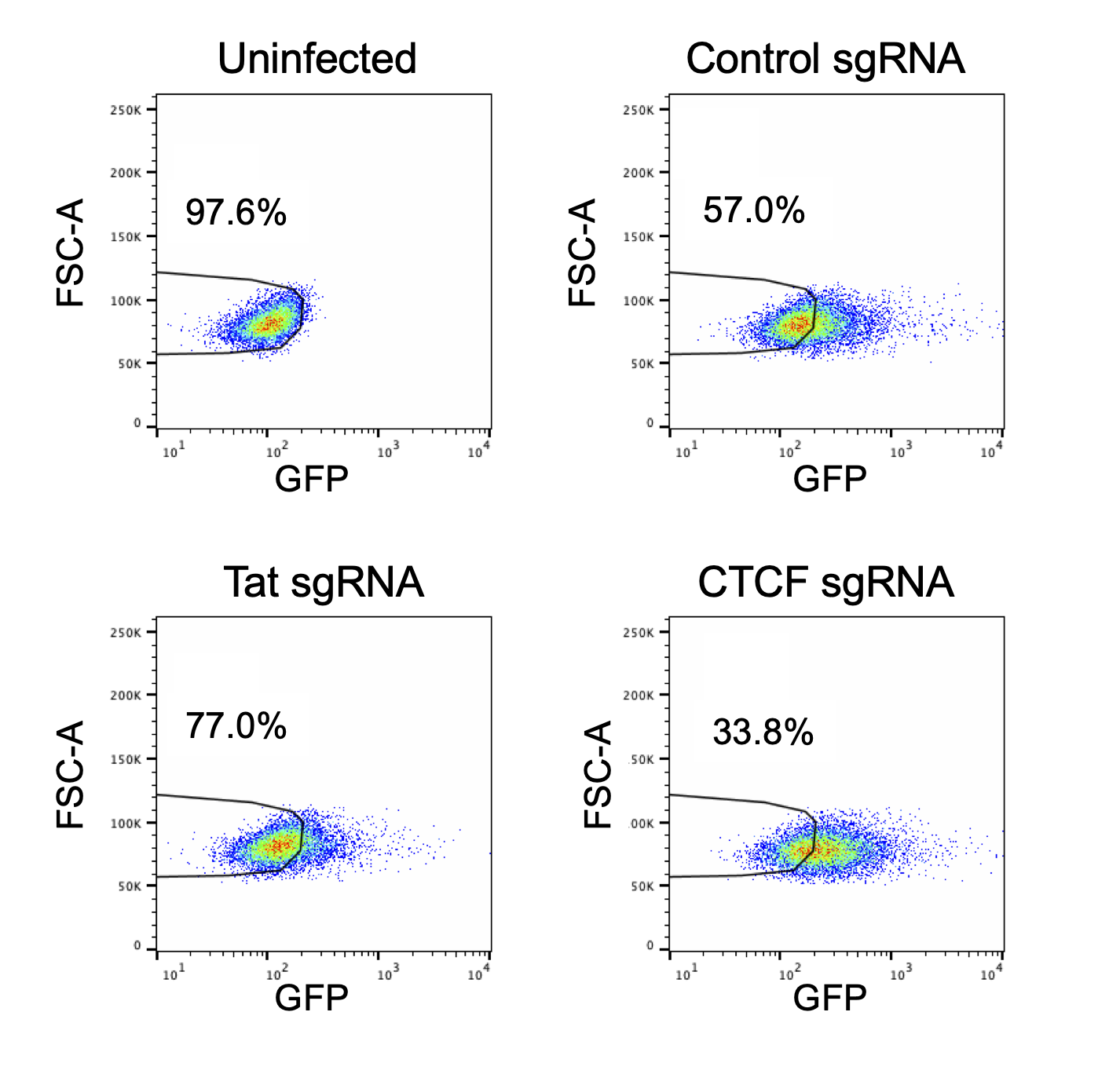

Supplement: S6 Fig — Underlying flow cytometry and gating for Fig 7F is shown. (TIF) [file ppat.1009346.s006.tif]
