# Supplementary material for: Epigenomic characterization of latent HIV infection identifies latency regulating transcription factors
Source: PLoS Pathog. 2021 Feb 26;17(2):e1009346. doi: 10.1371/journal.ppat.1009346 (PMC7946360; doi:10.1371/journal.ppat.1009346)
Supplement: S1 Table — For each experimental comparison, the number of cellular genes associated with a significant change for its RNA transcript and/or an ATACseq peak (fold change>1.5, Padj<0.1), were calculated. Statistical enrichment of ATACseq peak changes in genes with changing transcript levels was calculated using Fisher’s exact test (lower right panel). (DOC) [file ppat.1009346.s007.doc]

*S1 Table Enrichment of ATACseq peaks in differentially expressed genes.*

For each experimental comparison, the number of cellular genes associated with a significant change for its RNA transcript and/or an ATACseq peak (fold change>1.5, P_adj_<0.1), were calculated. Statistical enrichment of ATACseq peak changes in genes with changing transcript levels was calculated using Fisher’s exact test (lower right panel).

|  | **Latent vs active infection** | | |
| --- | --- | --- | --- |
|  |  | RNA transcript | |
|  |  | No Change | Different |
| ATACseq peak | No Change | 8773 | 2436 |
|  | Different | 1170 | 471 |
|  |  |  | P=8.17x10^-10^ |

|  | **AZD5582 vs DMSO** | | |
| --- | --- | --- | --- |
|  |  | RNA transcript | |
|  |  | No Change | Different |
| ATACseq peak | No Change | 7738 | 1858 |
|  | Different | 2310 | 833 |
|  |  |  | P=2.2x10^-16^ |

|  | **Vorinostat vs DMSO** | | |
| --- | --- | --- | --- |
|  |  | RNA transcript | |
|  |  | No Change | Different |
| ATACseq peak | No Change | 9734 | 2296 |
|  | Different | 526 | 158 |
|  |  |  | P=0.01104 |

|  | **Prostratin vs DMSO** | | |
| --- | --- | --- | --- |
|  |  | RNA transcript | |
|  |  | No Change | Different |
| ATACseq peak | No Change | 4110 | 1655 |
|  | Different | 4583 | 2390 |
|  |  |  | P=2.2x10^-16^ |
