# Supplementary material for: Epigenomic characterization of latent HIV infection identifies latency regulating transcription factors
Source: PLoS Pathog. 2021 Feb 26;17(2):e1009346. doi: 10.1371/journal.ppat.1009346 (PMC7946360; doi:10.1371/journal.ppat.1009346)
Supplement: S2 Table — Genes exhibiting a significant change to both transcript level and accessibility in actively infected cells (GFP+) vs latently infected cells (GFP-) are shown (fold change>1.5, Padj<0.1). Increased RNA levels or accessibility in actively infected cells (GFP+) are designated as changing in the “up” direction. (DOC) [file ppat.1009346.s008.doc]

| **Gene** | **Access Dir** | **ATAC peak Log2MeanFC** | **RNA Dir** | **RNA Log2FC** |
| --- | --- | --- | --- | --- |
| SRGAP3 | up | 3.814108873 | up | 2.053890499 |
| DACT2 | up | 3.603084918 | up | 6.301406374 |
| EXOC6B | mixed | 3.582232143 | up | 0.96660431 |
| TTLL12 | up | 3.572169171 | down | -1.417045036 |
| C15orf53 | up | 3.331203156 | up | 6.516487912 |
| KCND3 | up | 3.312545752 | up | 6.347077427 |
| NEO1 | up | 3.186641774 | up | 1.539744646 |
| ANKRD24 | up | 3.186635425 | up | 5.668119367 |
| CLPB | up | 3.102463393 | up | 1.765586772 |
| PLCG2 | up | 3.071563733 | up | 4.071622562 |
| UBN2 | up | 3.028464262 | up | 0.967866885 |
| CA10 | up | 3.019898532 | up | 7.752767964 |
| ANKRD28 | up | 2.993040563 | down | -0.877881336 |
| CCT6B | up | 2.989908408 | down | -2.513894153 |
| ERCC6 | up | 2.967568328 | up | 0.877374517 |
| BEND3 | up | 2.848730741 | up | 1.852790064 |
| ISPD | up | 2.778131382 | down | -5.786485339 |
| SMCO4 | up | 2.755281058 | up | 2.129228354 |
| RALGAPB | up | 2.724861801 | up | 0.596304723 |
| PLEKHG3 | up | 2.674855992 | up | 2.839612858 |
| HRH4 | up | 2.672697287 | down | -7.898511889 |
| CHN2 | up | 2.669154462 | up | 2.954491292 |
| AFAP1 | mixed | 2.649438222 | up | 0.744823231 |
| ENTHD1 | up | 2.642571239 | up | 6.471173662 |
| SOGA3 | up | 2.603679964 | up | 1.473501268 |
| KIAA0408 | up | 2.603679964 | up | 0.816550901 |
| NRIP3 | up | 2.589938631 | up | 6.29406726 |
| CADM1 | mixed | 2.581463795 | up | 3.263440924 |
| CHDH | up | 2.557184063 | up | 2.352774135 |
| BCAR3 | up | 2.498296612 | up | 8.442873658 |
| HNRNPLL | up | 2.497268002 | up | 0.894919723 |
| CHRM3 | up | 2.479338593 | up | 3.600014742 |
| IL17RB | up | 2.476078676 | up | 3.207625442 |
| ITGB1 | up | 2.426704844 | up | 0.948179138 |
| SNAP25 | up | 2.424202244 | down | -6.584923983 |
| GPR35 | up | 2.421262889 | up | 2.381614337 |
| SLC24A1 | up | 2.385909322 | up | 0.77950764 |
| STXBP4 | up | 2.371879569 | up | 1.879931577 |
| MRC2 | up | 2.369518165 | up | 1.532998098 |
| CENPN | up | 2.362805597 | up | 0.635284807 |
| CMIP | mixed | 2.353914243 | up | 1.245919828 |
| CCR4 | up | 2.349802047 | up | 1.801102952 |
| MAML1 | up | 2.348700292 | down | -1.069792414 |
| TSKU | up | 2.344205432 | up | 6.38832779 |
| CCDC86 | up | 2.340540487 | up | 0.817158737 |
| LIMA1 | up | 2.337115266 | up | 1.908733418 |
| GUSBP11 | up | 2.324058331 | down | -0.648746157 |
| GRB10 | up | 2.31242611 | down | -1.675733041 |
| TLK2 | mixed | 2.305497854 | down | -0.678698267 |
| TRRAP | up | 2.277014158 | up | 0.822877495 |
| TLCD2 | up | 2.264577242 | up | 2.288654497 |
| RYR1 | up | 2.248819779 | up | 5.343688917 |
| PGLYRP2 | up | 2.244872523 | up | 2.544076295 |
| EXT1 | mixed | 2.222650127 | up | 2.180200044 |
| HSPA1L | up | 2.218405228 | down | -0.748194775 |
| RPS6KA2 | up | 2.206089317 | down | -0.865883447 |
| LINC00565 | up | 2.198338998 | up | 6.835859932 |
| PPARG | up | 2.1681302 | down | -6.449122546 |
| TRAF3IP2-AS1 | up | 2.15891771 | up | 0.656462489 |
| EDN3 | up | 2.15600315 | up | 2.427251859 |
| ECSCR | up | 2.155653553 | up | 3.403621101 |
| RAB37 | up | 2.149330222 | up | 0.757126757 |
| USP6NL | up | 2.111693559 | down | -1.158614353 |
| HHAT | up | 2.103565405 | down | -0.902266541 |
| LOXL1 | up | 2.099384745 | down | -0.894587534 |
| BHLHE40-AS1 | up | 2.090124968 | up | 1.433663763 |
| PPP1R12B | up | 2.08390012 | up | 0.657394671 |
| SPRED2 | up | 2.082051599 | up | 1.143628611 |
| OPRL1 | up | 2.076430678 | down | -2.832379045 |
| LRRC47 | up | 2.076142892 | down | -3.806871405 |
| DRAM1 | up | 2.059400681 | up | 0.686693994 |
| GPR137B | up | 2.045229441 | up | 1.357488905 |
| ATXN1 | up | 2.044578356 | up | 0.881650899 |
| CIB2 | up | 2.041555056 | down | -2.454385951 |
| PRR5L | up | 2.01928936 | up | 3.211568994 |
| NUGGC | up | 2.018840127 | up | 7.232620696 |
| RBM20 | up | 2.015657622 | up | 6.592855685 |
| PARD3B | mixed | 2.004461508 | up | 6.630234014 |
| RUNX1 | up | 1.992638115 | down | -1.305626906 |
| CDPF1 | up | 1.990701297 | down | -0.679866976 |
| ZDHHC14 | up | 1.990691727 | up | 1.009636946 |
| CDR2 | up | 1.98555564 | down | -1.159550146 |
| IL4 | up | 1.975508606 | up | 3.666221859 |
| TIAM2 | up | 1.967746839 | up | 1.687362158 |
| PTPRO | up | 1.958975273 | down | -0.643411902 |
| DBH | up | 1.958431583 | up | 1.181829684 |
| ADAM19 | up | 1.939362754 | up | 1.448179264 |
| OTUD7A | up | 1.933317927 | down | -2.564631959 |
| EGOT | up | 1.929261523 | up | 6.896913406 |
| SPATA5 | up | 1.92539193 | up | 0.647095523 |
| RAD9B | up | 1.923652212 | down | -3.052502882 |
| CCSAP | up | 1.918966014 | down | -0.895250206 |
| CCR8 | up | 1.914910524 | up | 7.017316833 |
| CAPN12 | up | 1.901354423 | up | 1.902080804 |
| MORN2 | up | 1.895631901 | down | -0.975659448 |
| NCAPH | up | 1.894510885 | up | 2.039964214 |
| SOS2 | up | 1.889717828 | up | 1.774053187 |
| USP13 | up | 1.884413641 | down | -0.674649752 |
| ARMCX4 | up | 1.87917106 | up | 7.515267852 |
| PLB1 | up | 1.877286417 | up | 2.89210617 |
| KRT1 | up | 1.868057445 | up | 2.799550497 |
| UTRN | up | 1.866865764 | up | 0.885459114 |
| UPF1 | up | 1.862551905 | down | -1.580192607 |
| TMCC3 | up | 1.861261444 | down | -7.539865153 |
| ABCC4 | up | 1.860112773 | down | -1.595519108 |
| CYTH3 | up | 1.856360094 | up | 1.673133217 |
| BCL2L14 | up | 1.851131052 | up | 6.435125429 |
| LRP6 | up | 1.851131052 | up | 3.334151114 |
| SLCO3A1 | up | 1.834547256 | down | -1.845825784 |
| SLC9A5 | up | 1.830922088 | down | -1.175281353 |
| NHSL2 | up | 1.824850305 | up | 8.214110919 |
| MYOF | up | 1.823532056 | down | -6.327280353 |
| ZNF365 | up | 1.801981414 | up | 2.995689638 |
| ARHGEF18 | up | 1.799628522 | down | -1.801420102 |
| TRERF1 | up | 1.771865804 | up | 0.648233795 |
| UST | up | 1.770027929 | down | -1.065364363 |
| C5orf66 | up | 1.762014151 | up | 7.123002798 |
| CENPP | up | 1.760576477 | up | 1.627842303 |
| SLX4IP | up | 1.743968945 | up | 1.022863887 |
| KIAA0825 | up | 1.740006336 | down | -1.887111638 |
| C16orf45 | up | 1.733250437 | down | -1.058211745 |
| TEAD1 | mixed | 1.732197665 | down | -7.983584708 |
| NEK6 | up | 1.725376288 | up | 1.437371543 |
| FRMD4A | up | 1.719458263 | up | 3.374078582 |
| HOMER2 | up | 1.68977045 | up | 1.974476384 |
| LMNA | up | 1.684902674 | up | 1.385151515 |
| AKT3 | up | 1.68329685 | up | 0.858621781 |
| KRTAP5-AS1 | up | 1.682983386 | up | 6.801900178 |
| ESYT2 | up | 1.680905782 | down | -2.347578247 |
| KIF5A | up | 1.678962649 | down | -2.181080777 |
| AHNAK | up | 1.673372008 | up | 0.926119151 |
| CIITA | up | 1.673087886 | up | 1.048941466 |
| CRMP1 | up | 1.667251982 | up | 0.806901356 |
| PCBP3 | up | 1.655256403 | up | 1.538418095 |
| DNAI2 | up | 1.651808466 | up | 6.838142285 |
| CD84 | up | 1.647862449 | up | 0.945641878 |
| CDYL | up | 1.634564836 | down | -1.027664706 |
| SSH1 | up | 1.632383783 | up | 0.710872721 |
| LGR6 | up | 1.631591216 | up | 6.870642332 |
| TNRC18 | up | 1.619519769 | down | -1.235160674 |
| THBS1 | up | 1.60971028 | down | -5.789787447 |
| SNED1 | up | 1.602132384 | down | -0.79096159 |
| PHKA1 | up | 1.601703918 | up | 8.563576524 |
| NLN | mixed | 1.598951595 | up | 0.709903616 |
| EDARADD | mixed | 1.596560802 | up | 3.501929725 |
| CCR6 | up | 1.594490348 | up | 1.046097982 |
| KCNK1 | up | 1.578414618 | up | 6.630426008 |
| AIM2 | up | 1.576791286 | down | -1.250712355 |
| ZBTB16 | up | 1.572365777 | up | 1.106549983 |
| SRM | up | 1.568796248 | down | -2.483245032 |
| SPTY2D1 | up | 1.564545356 | up | 1.001178815 |
| MICAL2 | up | 1.562705818 | up | 1.663180702 |
| DUS2 | up | 1.55699008 | up | 0.600719095 |
| MATN2 | up | 1.545628278 | down | -6.005213316 |
| LRRC2 | up | 1.545151887 | up | 2.968522991 |
| B4GALT5 | up | 1.542131058 | up | 1.820773109 |
| ST7 | up | 1.53628592 | up | 2.265065478 |
| ADPGK-AS1 | up | 1.523093782 | up | 5.984610626 |
| PPP1R26-AS1 | up | 1.522017594 | up | 6.989780007 |
| FAM107B | up | 1.517022759 | down | -0.807533498 |
| PLEKHA5 | up | 1.515757295 | up | 2.776081111 |
| RCBTB2 | up | 1.51295544 | up | 0.616904353 |
| TNFRSF4 | up | 1.509879056 | up | 0.910074243 |
| SH3PXD2A | up | 1.493319977 | up | 1.725845725 |
| PIWIL2 | up | 1.487220069 | down | -2.242416332 |
| RANBP3 | up | 1.486339168 | up | 0.651708102 |
| TCN1 | up | 1.479704921 | up | 6.629162253 |
| SGSM2 | up | 1.479316797 | down | -2.669165521 |
| LINC00511 | up | 1.475325663 | up | 1.373808562 |
| BLM | up | 1.470712115 | down | -0.595258858 |
| REC8 | up | 1.467567129 | up | 0.809392213 |
| NRP1 | up | 1.465918036 | down | -1.444708326 |
| SMAD3 | up | 1.465106707 | down | -0.861735654 |
| FRMD4B | up | 1.465000687 | up | 1.045675027 |
| ANKH | up | 1.463641348 | down | -2.131268704 |
| PAQR6 | up | 1.462933125 | up | 6.958601627 |
| FILIP1 | up | 1.455794525 | up | 2.761648625 |
| TLE3 | up | 1.454689585 | down | -1.099785567 |
| GALM | up | 1.453557085 | up | 0.623427249 |
| DAPK2 | up | 1.45139691 | up | 2.710597115 |
| ARHGAP10 | up | 1.440649626 | down | -1.030624836 |
| SIK3 | up | 1.438860634 | down | -2.80792494 |
| MMP2 | up | 1.432257879 | up | 4.486666397 |
| RORA | up | 1.423253082 | up | 0.673463866 |
| EIF4E3 | up | 1.423220235 | down | -0.696309262 |
| CD200R1 | up | 1.421870129 | up | 1.442704185 |
| FMNL2 | up | 1.418157698 | down | -6.670429899 |
| GPR157 | up | 1.417162492 | down | -0.990492564 |
| GPATCH2 | up | 1.415056666 | up | 0.823502975 |
| SLC7A5 | up | 1.412121049 | down | -0.903016936 |
| SOGA1 | up | 1.40929453 | down | -1.399806681 |
| GRK6 | up | 1.406564152 | down | -1.03007043 |
| CD70 | up | 1.400977669 | down | -1.42346462 |
| SLC35E1 | up | 1.395162575 | down | -1.147937604 |
| CBFB | up | 1.394553195 | down | -1.802032736 |
| PMF1-BGLAP | up | 1.390222711 | up | 1.115981276 |
| ABCA2 | up | 1.389339725 | down | -1.264076724 |
| RAB27B | mixed | 1.384798698 | up | 8.002763495 |
| TBXAS1 | up | 1.381110604 | up | 2.089510549 |
| SLC1A4 | up | 1.374153618 | down | -1.231173093 |
| ADAM23 | up | 1.37163045 | up | 1.696803868 |
| RDX | up | 1.366019006 | down | -0.688945244 |
| PACS1 | up | 1.363569616 | down | -1.269115139 |
| OTOF | up | 1.360452404 | down | -7.000557035 |
| RXRA | up | 1.360437602 | up | 2.788598229 |
| DOCK9 | up | 1.354784742 | down | -0.785996765 |
| DNAJC18 | up | 1.35380073 | up | 1.639278666 |
| WWTR1 | mixed | 1.352465473 | down | -1.112239282 |
| ASCC3 | up | 1.340502266 | up | 0.73051716 |
| MAP1B | up | 1.338683143 | down | -4.956102169 |
| TMCC2 | up | 1.336644365 | up | 6.250352507 |
| LINC01307 | up | 1.335339259 | down | -7.240092815 |
| POMT2 | up | 1.330098516 | down | -1.41636276 |
| GPR55 | up | 1.325852345 | up | 1.219685718 |
| KLHL11 | up | 1.324968684 | up | 1.017691231 |
| CUBN | up | 1.319470641 | up | 1.090535956 |
| APPL2 | up | 1.315521547 | down | -1.571618332 |
| ELAVL1 | up | 1.311293061 | down | -1.892184364 |
| SATB1-AS1 | mixed | 1.296419221 | down | -0.97052564 |
| PTGDR2 | up | 1.290857436 | up | 3.668589516 |
| IL9R | up | 1.290764316 | up | 2.961017554 |
| CYP1B1 | up | 1.289432954 | up | 2.689683226 |
| RNF19A | up | 1.285428068 | up | 0.627509156 |
| ARL17B | up | 1.28459084 | up | 0.800176606 |
| FSIP1 | up | 1.282230357 | down | -2.68131492 |
| TNC | up | 1.279029539 | down | -8.140226742 |
| TRAPPC10 | up | 1.274160302 | down | -1.498387342 |
| ZSWIM4 | up | 1.271130181 | down | -2.428836135 |
| MAPK1 | up | 1.269160727 | down | -2.394815722 |
| OTUB2 | up | 1.266906465 | down | -1.138686207 |
| FAM167A | up | 1.261912027 | up | 7.944331171 |
| ID1 | up | 1.258785898 | down | -6.239732917 |
| CLMN | up | 1.253096252 | up | 8.215251652 |
| TCF20 | up | 1.248883339 | up | 0.956336589 |
| RFX2 | up | 1.246391745 | down | -1.052780617 |
| CKAP2L | up | 1.242714695 | down | -5.241003419 |
| WDR27 | up | 1.238280418 | up | 0.849365249 |
| GK | up | 1.237371453 | up | 0.853884084 |
| FOXB1 | up | 1.230336101 | up | 7.251634963 |
| RASGRP3 | up | 1.223701824 | up | 0.690059802 |
| NEK10 | up | 1.22256858 | down | -6.398980456 |
| IL12RB2 | up | 1.221939203 | up | 4.093500963 |
| SLAMF8 | up | 1.221897356 | up | 6.299618599 |
| SPEN | up | 1.212340383 | down | -0.669376495 |
| NF1 | up | 1.20685401 | up | 0.779614543 |
| FAM47E-STBD1 | up | 1.204610901 | up | 1.196003081 |
| SH3RF1 | up | 1.19629238 | up | 2.917048399 |
| HCAR1 | up | 1.16913165 | up | 7.869606619 |
| FGGY | up | 1.164183661 | up | 1.739584367 |
| EXOSC4 | up | 1.160399287 | up | 0.63263774 |
| TMPO-AS1 | up | 1.157933801 | up | 1.100604733 |
| LTBP2 | up | 1.155830081 | up | 1.488975977 |
| RGS9 | up | 1.154789735 | up | 1.952457816 |
| ASPH | up | 1.154475877 | up | 0.831142616 |
| CELSR1 | up | 1.145280862 | down | -7.18509002 |
| CDH1 | up | 1.132226221 | up | 7.738609414 |
| TRIM2 | up | 1.131104268 | up | 2.153740067 |
| CDH23 | up | 1.129614917 | up | 2.661396708 |
| SUN2 | up | 1.12937976 | down | -2.661871984 |
| TRIP13 | up | 1.120626344 | down | -1.267764593 |
| LINC00672 | up | 1.119299532 | down | -2.553315618 |
| AK9 | up | 1.098042122 | up | 0.78509227 |
| MACF1 | up | 1.096211954 | up | 0.806115013 |
| SLAIN1 | up | 1.087140036 | down | -0.606443694 |
| GGA3 | up | 1.084453478 | down | -0.619055246 |
| FAM53B | up | 1.082580313 | down | -0.913664622 |
| FBXO10 | up | 1.069180308 | up | 0.630425355 |
| GNAO1 | up | 1.065515538 | up | 3.930111028 |
| NOD2 | up | 1.062964839 | up | 2.247325648 |
| IFNG-AS1 | up | 1.05958166 | up | 8.163916653 |
| TUBA8 | up | 1.059128271 | up | 4.4835631 |
| CASP9 | up | 1.058303025 | down | -0.678582992 |
| ZBTB7A | up | 1.058289693 | down | -2.281474913 |
| TRIB1 | up | 1.05081232 | down | -1.328925797 |
| BMP1 | up | 1.048698649 | down | -2.790215691 |
| MED12L | up | 1.043303156 | up | 5.150107645 |
| CLSTN1 | up | 1.041937463 | down | -1.720041212 |
| ATF1 | up | 1.040883567 | down | -0.652833141 |
| ANPEP | up | 1.038192224 | up | 3.651782152 |
| TTC39C | up | 1.030672496 | down | -0.798625216 |
| IL18RAP | up | 1.027488452 | up | 1.640559429 |
| NCAPG2 | up | 1.02367746 | up | 1.267300098 |
| HTR2B | up | 1.022022781 | up | 4.667749977 |
| HLA-DRA | up | 1.021053354 | up | 2.739125003 |
| ME3 | up | 1.016640643 | up | 6.929654516 |
| SH3TC1 | up | 1.001524847 | up | 0.614077971 |
| PLEK | up | 0.999864616 | up | 8.525348652 |
| GARNL3 | up | 0.995187406 | up | 3.118802386 |
| FBXO45 | up | 0.985580733 | up | 0.766924184 |
| WDR53 | up | 0.985580733 | up | 0.60705144 |
| EFHD2 | up | 0.973797754 | down | -0.928986372 |
| VDR | up | 0.970188649 | up | 1.150918277 |
| PPCDC | up | 0.9577061 | up | 0.598150832 |
| RGL1 | up | 0.957220251 | up | 0.70378028 |
| C10orf105 | up | 0.956417521 | down | -7.563528797 |
| EFCAB11 | up | 0.952823683 | down | -3.029573756 |
| INTS1 | up | 0.952595306 | down | -0.700625953 |
| CDC6 | up | 0.951465796 | down | -3.798525805 |
| TTC9 | up | 0.947700371 | down | -1.300831997 |
| SLC29A4 | up | 0.946751528 | up | 3.501792278 |
| RMND5A | up | 0.944445343 | down | -0.770378946 |
| CRIM1 | up | 0.940771905 | up | 2.709728775 |
| CTSA | up | 0.938881883 | up | 0.683633951 |
| LPCAT2 | up | 0.927327868 | up | 1.72834876 |
| MTHFD1L | up | 0.927225108 | down | -1.634746868 |
| FASLG | up | 0.91669516 | up | 2.3702081 |
| HLA-DPA1 | up | 0.909145574 | up | 1.013246876 |
| ETFB | up | 0.906374513 | up | 0.838886452 |
| GLIS2 | up | 0.904483308 | up | 2.761510699 |
| KALRN | up | 0.901988839 | down | -9.27583212 |
| INSR | up | 0.898037429 | up | 1.160461838 |
| IQSEC2 | up | 0.88816861 | up | 5.686202365 |
| BMP4 | up | 0.887742919 | up | 8.524398678 |
| MMP24 | up | 0.887662329 | up | 2.316874345 |
| RELB | up | 0.861812191 | down | -0.622394382 |
| VOPP1 | up | 0.854881633 | down | -0.587149265 |
| B3GALNT2 | up | 0.847083123 | up | 0.688671595 |
| RFPL2 | up | 0.839259608 | up | 5.516138796 |
| PTPN13 | up | 0.813417967 | up | 6.202783445 |
| LRRC20 | up | 0.809124476 | down | -1.063762879 |
| NMUR1 | up | 0.804874941 | up | 4.344622646 |
| TRPS1 | up | 0.803253333 | down | -0.70275459 |
| SULT2B1 | up | 0.793302168 | up | 5.253374219 |
| BLZF1 | up | 0.781768085 | up | 0.738138778 |
| KAT6B | up | 0.780478648 | up | 0.593949198 |
| ST6GALNAC3 | up | 0.779014004 | up | 7.835889816 |
| IFNL1 | up | 0.772033182 | up | 3.929056596 |
| TP53I11 | up | 0.767964359 | up | 0.91269859 |
| SPNS3 | up | 0.766470785 | up | 0.886010835 |
| SIGLEC6 | up | 0.76318233 | up | 6.737591886 |
| HLA-DRB1 | up | 0.758512976 | up | 2.135720697 |
| TFEB | up | 0.752224595 | down | -1.073435776 |
| HAR1B | up | 0.745508514 | up | 2.334963742 |
| HAR1A | up | 0.745508514 | up | 0.708092839 |
| TMEM63C | up | 0.744593224 | up | 5.157289697 |
| PPP2R2D | up | 0.703765593 | down | -1.99757568 |
| WDR11 | up | 0.702926328 | up | 0.624423025 |
| CCL5 | up | 0.695207752 | up | 0.714811664 |
| DOCK1 | up | 0.685947795 | up | 7.431262552 |
| TPRG1 | up | 0.685283394 | up | 0.720362915 |
| GNA15 | up | 0.684995398 | up | 1.322771189 |
| NKPD1 | up | 0.671587518 | down | -1.3646072 |
| CLU | up | 0.589677624 | up | 2.884365711 |
| FBLN1 | down | -0.719662962 | down | -6.286493403 |
| CHIT1 | down | -0.731903266 | up | 8.060538628 |
| EFNA4 | down | -0.75374378 | up | 1.69609242 |
| PARPBP | down | -0.760611527 | down | -0.884439014 |
| KIF23 | down | -0.760636615 | up | 7.929299841 |
| JUP | down | -0.795659452 | up | 7.275423919 |
| KLRK1 | down | -0.822488758 | up | 0.802252202 |
| TTC8 | down | -0.83816535 | up | 2.225946833 |
| DSC1 | down | -0.844549684 | down | -1.784392238 |
| DISC1 | down | -0.873427489 | up | 0.992661592 |
| PTPRA | down | -0.875859106 | down | -1.991337508 |
| DPY19L1 | mixed | -0.954493593 | down | -1.230090031 |
| CAMSAP2 | down | -0.963922262 | down | -2.16488896 |
| TMEM62 | down | -0.993600007 | up | 0.871737552 |
| TXLNB | down | -1.013999178 | down | -3.095029125 |
| ARHGAP21 | down | -1.024653211 | down | -2.245485712 |
| ZNF736 | down | -1.038410346 | down | -0.593340039 |
| TAF5 | down | -1.049681244 | up | 0.789323197 |
| LAMB1 | down | -1.065712305 | down | -3.327445231 |
| TPST2 | down | -1.070367902 | up | 0.589280192 |
| KLRB1 | down | -1.121276218 | up | 2.800683945 |
| GCNT2 | down | -1.138137943 | up | 1.120118022 |
| MAPT | down | -1.142325164 | up | 6.514210556 |
| ITGA2 | down | -1.143989006 | up | 1.026178356 |
| PLCXD2 | down | -1.159325097 | up | 0.630305799 |
| RAPGEF2 | down | -1.166542166 | up | 0.780996208 |
| ABHD12 | down | -1.190693191 | down | -1.944658289 |
| DNAJC1 | down | -1.203448991 | down | -0.776710437 |
| NCALD | down | -1.240556233 | up | 3.082262374 |
| WDPCP | down | -1.247855371 | down | -1.093541631 |
| NRIP1 | down | -1.278148993 | down | -2.37473772 |
| KIAA0895 | down | -1.286919491 | down | -1.500062023 |
| PHEX | down | -1.297313902 | up | 6.702937791 |
| DMXL2 | down | -1.313039215 | up | 9.493687071 |
| PHTF2 | mixed | -1.329923179 | up | 1.118481117 |
| KLHL2 | down | -1.334832745 | down | -1.410792478 |
| ASB2 | down | -1.335409644 | up | 2.145285862 |
| KCNAB1 | down | -1.355737646 | down | -1.181269867 |
| WDFY4 | down | -1.368601046 | down | -0.820350399 |
| NDUFAF6 | down | -1.409009028 | down | -0.73470211 |
| RCC2 | down | -1.415799105 | down | -1.750000191 |
| YIPF6 | down | -1.428925405 | up | 0.719611385 |
| XPR1 | down | -1.435827269 | down | -0.777016842 |
| NAF1 | down | -1.437114558 | up | 0.937648429 |
| EPHB1 | down | -1.43917835 | up | 2.584081547 |
| PLK4 | down | -1.445931026 | up | 4.356663913 |
| STIM2 | down | -1.447175893 | down | -1.56487084 |
| AP3S2 | down | -1.452305488 | up | 0.641311782 |
| ENPP2 | down | -1.458279123 | down | -1.955309212 |
| MICAL3 | down | -1.461670902 | up | 1.741964154 |
| CDYL2 | down | -1.471199845 | up | 1.296836427 |
| EPS8 | down | -1.503811492 | down | -2.750954447 |
| NEK7 | down | -1.520973614 | down | -1.633335422 |
| EIF2AK3 | down | -1.543136379 | down | -1.87591252 |
| TBC1D1 | down | -1.546771742 | up | 0.710463005 |
| MPP6 | down | -1.581693436 | down | -0.776186709 |
| CAMK1D | down | -1.582994314 | down | -0.628612639 |
| C22orf34 | down | -1.583637 | down | -0.602247116 |
| FAM13A-AS1 | down | -1.585492703 | down | -2.415257431 |
| OGFRL1 | down | -1.587592424 | down | -2.969433358 |
| KANSL1L | down | -1.592156367 | down | -0.735437371 |
| DOK6 | down | -1.597469924 | up | 4.071564074 |
| C7orf26 | down | -1.618359289 | down | -1.599596982 |
| SEMA3A | down | -1.633470545 | down | -6.123358897 |
| CACHD1 | down | -1.650896066 | down | -8.999763439 |
| APBB2 | down | -1.651000668 | down | -3.846504343 |
| IMMP2L | down | -1.673238217 | up | 1.051565533 |
| LAMA2 | down | -1.6929555 | up | 7.201694813 |
| OSBPL1A | down | -1.700778382 | up | 5.044487415 |
| CASZ1 | down | -1.703897115 | up | 1.077829648 |
| CFI | down | -1.705828501 | down | -7.241540471 |
| CDC42EP4 | down | -1.730249221 | up | 2.435520378 |
| SNX16 | down | -1.812074515 | down | -0.63382506 |
| DIRC2 | down | -1.814636917 | up | 0.627640441 |
| SPC25 | down | -1.827116564 | up | 6.046415957 |
| FAM13A | down | -1.82741697 | down | -1.068512517 |
| ZBTB25 | down | -1.82878497 | up | 0.625911452 |
| FNIP2 | down | -1.831566445 | up | 0.599295934 |
| ADAMTS17 | down | -1.836580146 | down | -1.329303223 |
| GPAM | down | -1.850611534 | down | -0.701685396 |
| GSDMB | down | -1.850741376 | up | 1.424391187 |
| AK5 | down | -1.89100395 | down | -0.605442301 |
| C3orf52 | down | -1.891294949 | up | 0.67150245 |
| OSBPL6 | down | -1.899203641 | up | 8.544812723 |
| POU5F2 | down | -1.922056056 | up | 8.899426052 |
| MRVI1 | down | -1.923089307 | down | -7.241182348 |
| KIF18A | down | -1.926427038 | down | -2.964000332 |
| PACRGL | down | -1.930661446 | up | 1.440875988 |
| RNLS | down | -1.930837417 | up | 0.711047723 |
| OXR1 | down | -1.939698413 | down | -0.777405936 |
| SMAD5 | down | -1.97496428 | up | 2.880615124 |
| ZNF273 | down | -1.976878777 | up | 1.342770439 |
| DPYD | down | -2.000540596 | up | 0.614960161 |
| CTIF | down | -2.002428988 | up | 3.224832619 |
| CDS1 | down | -2.003882363 | down | -4.851144656 |
| CCNA2 | down | -2.017204278 | up | 3.469988045 |
| DIP2C | mixed | -2.0224093 | down | -1.543379498 |
| KCNQ1 | down | -2.029934704 | down | -2.540114218 |
| GALNT10 | down | -2.030631358 | down | -2.379093706 |
| FIZ1 | down | -2.057891669 | down | -2.145882888 |
| BAZ2B | down | -2.091479964 | up | 0.70951756 |
| UNC45B | down | -2.096786026 | up | 1.48768767 |
| CDC25B | down | -2.098474338 | down | -0.887686059 |
| SETD7 | down | -2.146513704 | up | 0.588484181 |
| TRAF6 | down | -2.156254554 | up | 0.665631889 |
| PCED1B | down | -2.228753323 | down | -0.666048751 |
| 1-Dec | down | -2.233754704 | up | 6.73796871 |
| PTPRK | down | -2.241752865 | down | -1.304338072 |
| CAB39L | down | -2.245366588 | up | 0.630831339 |
| EYS | down | -2.254382742 | up | 3.743145397 |
| CPEB3 | down | -2.285750664 | down | -2.288985587 |
| KIF26B | down | -2.288887633 | down | -5.709670215 |
| TRIO | down | -2.348317762 | up | 5.392492378 |
| SDK2 | down | -2.352903485 | down | -1.799735932 |
| NCOA2 | down | -2.357651653 | up | 0.635387122 |
| RALGPS1 | mixed | -2.375010092 | up | 5.895875517 |
| DLGAP1 | down | -2.426819251 | down | -6.584837341 |
| LINC00886 | down | -2.456809322 | down | -4.534003788 |
| ATG10 | down | -2.465146408 | up | 0.604767155 |
| HRH1 | mixed | -2.508136032 | up | 9.093352336 |
| DAZAP1 | mixed | -2.559478657 | down | -0.842650556 |
| TTC28 | down | -2.706108474 | down | -0.708163438 |
| MCF2L2 | down | -2.74442785 | up | 4.092781494 |
| IGF1R | down | -2.789041696 | down | -0.975645079 |
| DUSP19 | down | -2.793342471 | up | 1.286299402 |
| VAV2 | mixed | -2.820028501 | down | -2.073717687 |
| CRTAM | down | -3.102750406 | down | -7.540519035 |
| RBKS | down | -3.375566648 | down | -0.776965756 |
