# Supplementary material for: Epigenomic characterization of latent HIV infection identifies latency regulating transcription factors
Source: PLoS Pathog. 2021 Feb 26;17(2):e1009346. doi: 10.1371/journal.ppat.1009346 (PMC7946360; doi:10.1371/journal.ppat.1009346)
Supplement: S3 Table — HOMER TF motif enrichment analysis of differentially open peaks between actively infected cells (GFP+) and latently infected (GFP-) cells. Top 100 enriched motifs in peaks preferentially open in actively infected cells are shown. Target Sequences represent genomic sequences that have significantly elevated accessibility in actively infected cells. Background sequences represent all open chromatin regions in CD4 T cells. (DOC) [file ppat.1009346.s009.doc]

*S3 Table. TF motif enrichment in actively infected cells.*

HOMER TF motif enrichment analysis of differentially open peaks between actively infected cells (GFP+) and latently infected (GFP-) cells. Top 100 enriched motifs in peaks preferentially open in actively infected cells are shown. Target Sequences represent genomic sequences that have significantly elevated accessibility in latently infected cells. Background sequences represent all open chromatin regions in CD4 T cells.

| Motif Name | Consensus | P-value | % of Target Sequences with Motif | % of Background Sequences with Motif |
| --- | --- | --- | --- | --- |
| BATF(bZIP)/Th17-BATF-ChIP-Seq(GSE39756)/Homer | DATGASTCAT | 1e-449 | 37.09% | 12.04% |
| Atf3(bZIP)/GBM-ATF3-ChIP-Seq(GSE33912)/Homer | DATGASTCATHN | 1e-446 | 37.52% | 12.38% |
| AP-1(bZIP)/ThioMac-PU.1-ChIP-Seq(GSE21512)/Homer | VTGACTCATC | 1e-443 | 39.24% | 13.54% |
| Fra1(bZIP)/BT549-Fra1-ChIP-Seq(GSE46166)/Homer | NNATGASTCATH | 1e-441 | 34.27% | 10.51% |
| JunB(bZIP)/DendriticCells-Junb-ChIP-Seq(GSE36099)/Homer | RATGASTCAT | 1e-439 | 34.21% | 10.50% |
| RUNX1(Runt)/Jurkat-RUNX1-ChIP-Seq(GSE29180)/Homer | AAACCACARM | 1e-436 | 48.42% | 20.08% |
| Fra2(bZIP)/Striatum-Fra2-ChIP-Seq(GSE43429)/Homer | GGATGACTCATC | 1e-428 | 31.71% | 9.25% |
| Fosl2(bZIP)/3T3L1-Fosl2-ChIP-Seq(GSE56872)/Homer | NATGASTCABNN | 1e-395 | 25.97% | 6.69% |
| RUNX2(Runt)/PCa-RUNX2-ChIP-Seq(GSE33889)/Homer | NWAACCACADNN | 1e-375 | 41.60% | 16.72% |
| RUNX-AML(Runt)/CD4+-PolII-ChIP-Seq(Barski_et_al.)/Homer | GCTGTGGTTW | 1e-356 | 38.68% | 15.16% |
| RUNX(Runt)/HPC7-Runx1-ChIP-Seq(GSE22178)/Homer | SAAACCACAG | 1e-356 | 38.06% | 14.74% |
| Jun-AP1(bZIP)/K562-cJun-ChIP-Seq(GSE31477)/Homer | GATGASTCATCN | 1e-349 | 21.31% | 5.02% |
| TRPS1(Zf)/MCF7-TRPS1-ChIP-Seq(GSE107013)/Homer | AGATAAGANN | 1.00E-300 | 53.36% | 28.31% |
| GATA3(Zf)/iTreg-Gata3-ChIP-Seq(GSE20898)/Homer | AGATAASR | 1.00E-287 | 46.24% | 22.87% |
| SCL(bHLH)/HPC7-Scl-ChIP-Seq(GSE13511)/Homer | AVCAGCTG | 1.00E-248 | 73.25% | 49.88% |
| Gata6(Zf)/HUG1N-GATA6-ChIP-Seq(GSE51936)/Homer | YCTTATCTBN | 1.00E-234 | 33.61% | 14.99% |
| Gata4(Zf)/Heart-Gata4-ChIP-Seq(GSE35151)/Homer | NBWGATAAGR | 1.00E-234 | 35.53% | 16.39% |
| Nanog(Homeobox)/mES-Nanog-ChIP-Seq(GSE11724)/Homer | RGCCATTAAC | 1.00E-232 | 67.59% | 44.70% |
| Bach2(bZIP)/OCILy7-Bach2-ChIP-Seq(GSE44420)/Homer | TGCTGAGTCA | 1.00E-213 | 15.03% | 3.92% |
| Pitx1(Homeobox)/Chicken-Pitx1-ChIP-Seq(GSE38910)/Homer | TAATCCCN | 1.00E-213 | 66.85% | 44.94% |
| Smad3(MAD)/NPC-Smad3-ChIP-Seq(GSE36673)/Homer | TWGTCTGV | 1.00E-203 | 58.04% | 36.75% |
| Gata2(Zf)/K562-GATA2-ChIP-Seq(GSE18829)/Homer | BBCTTATCTS | 1.00E-198 | 26.79% | 11.31% |
| Tgif1(Homeobox)/mES-Tgif1-ChIP-Seq(GSE55404)/Homer | YTGWCADY | 1.00E-195 | 61.68% | 40.67% |
| Gata1(Zf)/K562-GATA1-ChIP-Seq(GSE18829)/Homer | SAGATAAGRV | 1.00E-192 | 24.91% | 10.22% |
| ERG(ETS)/VCaP-ERG-ChIP-Seq(GSE14097)/Homer | ACAGGAAGTG | 1.00E-190 | 52.40% | 32.19% |
| THRb(NR)/Liver-NR1A2-ChIP-Seq(GSE52613)/Homer | TRAGGTCA | 1.00E-185 | 63.21% | 42.75% |
| RARa(NR)/K562-RARa-ChIP-Seq(Encode)/Homer | TTGAMCTTTG | 1.00E-180 | 55.14% | 35.22% |
| ETV1(ETS)/GIST48-ETV1-ChIP-Seq(GSE22441)/Homer | AACCGGAAGT | 1.00E-180 | 48.16% | 28.89% |
| Tgif2(Homeobox)/mES-Tgif2-ChIP-Seq(GSE55404)/Homer | TGTCANYT | 1.00E-179 | 63.07% | 42.94% |
| AR-halfsite(NR)/LNCaP-AR-ChIP-Seq(GSE27824)/Homer | CCAGGAACAG | 1.00E-179 | 63.65% | 43.54% |
| Nkx6.1(Homeobox)/Islet-Nkx6.1-ChIP-Seq(GSE40975)/Homer | GKTAATGR | 1.00E-175 | 50.24% | 30.98% |
| Nkx3.1(Homeobox)/LNCaP-Nkx3.1-ChIP-Seq(GSE28264)/Homer | AAGCACTTAA | 1.00E-165 | 52.76% | 33.80% |
| Tbx5(T-box)/HL1-Tbx5.biotin-ChIP-Seq(GSE21529)/Homer | AGGTGTCA | 1.00E-165 | 64.07% | 44.77% |
| Ptf1a(bHLH)/Panc1-Ptf1a-ChIP-Seq(GSE47459)/Homer | ACAGCTGTTN | 1.00E-163 | 55.88% | 36.84% |
| Hoxa9(Homeobox)/ChickenMSG-Hoxa9.Flag-ChIP-Seq(GSE86088)/Homer | RGCAATNAAA | 1.00E-155 | 51.58% | 33.30% |
| Fli1(ETS)/CD8-FLI-ChIP-Seq(GSE20898)/Homer | NRYTTCCGGH | 1.00E-153 | 42.64% | 25.43% |
| Foxo1(Forkhead)/RAW-Foxo1-ChIP-Seq(Fan_et_al.)/Homer | CTGTTTAC | 1.00E-149 | 45.26% | 27.91% |
| CRX(Homeobox)/Retina-Crx-ChIP-Seq(GSE20012)/Homer | GCTAATCC | 1.00E-149 | 49.20% | 31.44% |
| Zac1(Zf)/Neuro2A-Plagl1-ChIP-Seq(GSE75942)/Homer | HAWGRGGCCM | 1.00E-149 | 52.20% | 34.19% |
| PR(NR)/T47D-PR-ChIP-Seq(GSE31130)/Homer | VAGRACAKNCTGTBC | 1.00E-148 | 47.40% | 29.88% |
| MafA(bZIP)/Islet-MafA-ChIP-Seq(GSE30298)/Homer | TGCTGACTCA | 1.00E-146 | 26.63% | 12.92% |
| Nkx2.1(Homeobox)/LungAC-Nkx2.1-ChIP-Seq(GSE43252)/Homer | RSCACTYRAG | 1.00E-145 | 58.50% | 40.42% |
| ETV4(ETS)/HepG2-ETV4-ChIP-Seq(ENCODE)/Homer | ACCGGAAGTG | 1.00E-144 | 41.68% | 25.05% |
| Eomes(T-box)/H9-Eomes-ChIP-Seq(GSE26097)/Homer | ATTAACACCT | 1.00E-144 | 48.30% | 30.93% |
| Meis1(Homeobox)/MastCells-Meis1-ChIP-Seq(GSE48085)/Homer | VGCTGWCAVB | 1.00E-143 | 44.10% | 27.20% |
| EHF(ETS)/LoVo-EHF-ChIP-Seq(GSE49402)/Homer | AVCAGGAAGT | 1.00E-142 | 42.14% | 25.58% |
| Nkx2.5(Homeobox)/HL1-Nkx2.5.biotin-ChIP-Seq(GSE21529)/Homer | RRSCACTYAA | 1.00E-140 | 51.76% | 34.33% |
| Bapx1(Homeobox)/VertebralCol-Bapx1-ChIP-Seq(GSE36672)/Homer | TTRAGTGSYK | 1.00E-138 | 52.12% | 34.74% |
| Hoxd11(Homeobox)/ChickenMSG-Hoxd11.Flag-ChIP-Seq(GSE86088)/Homer | VGCCATAAAA | 1.00E-137 | 47.28% | 30.36% |
| Hoxa13(Homeobox)/ChickenMSG-Hoxa13.Flag-ChIP-Seq(GSE86088)/Homer | CYHATAAAAN | 1.00E-134 | 48.10% | 31.31% |
| ETS1(ETS)/Jurkat-ETS1-ChIP-Seq(GSE17954)/Homer | ACAGGAAGTG | 1.00E-133 | 41.52% | 25.53% |
| BMAL1(bHLH)/Liver-Bmal1-ChIP-Seq(GSE39860)/Homer | GNCACGTG | 1.00E-132 | 41.12% | 25.25% |
| Etv2(ETS)/ES-ER71-ChIP-Seq(GSE59402)/Homer | NNAYTTCCTGHN | 1.00E-131 | 39.32% | 23.75% |
| Erra(NR)/HepG2-Erra-ChIP-Seq(GSE31477)/Homer | CAAAGGTCAG | 1.00E-130 | 45.28% | 28.99% |
| Sox10(HMG)/SciaticNerve-Sox3-ChIP-Seq(GSE35132)/Homer | CCWTTGTYYB | 1.00E-129 | 37.96% | 22.67% |
| Smad4(MAD)/ESC-SMAD4-ChIP-Seq(GSE29422)/Homer | VBSYGTCTGG | 1.00E-128 | 38.72% | 23.40% |
| Hoxa11(Homeobox)/ChickenMSG-Hoxa11.Flag-ChIP-Seq(GSE86088)/Homer | TTTTATGGCM | 1.00E-128 | 45.92% | 29.71% |
| PU.1-IRF(ETS:IRF)/Bcell-PU.1-ChIP-Seq(GSE21512)/Homer | MGGAAGTGAAAC | 1.00E-126 | 38.70% | 23.50% |
| COUP-TFII(NR)/K562-NR2F1-ChIP-Seq(Encode)/Homer | GKBCARAGGTCA | 1.00E-125 | 35.25% | 20.61% |
| TCF4(bHLH)/SHSY5Y-TCF4-ChIP-Seq(GSE96915)/Homer | SMCATCTGKH | 1.00E-125 | 37.92% | 22.88% |
| Ap4(bHLH)/AML-Tfap4-ChIP-Seq(GSE45738)/Homer | NAHCAGCTGD | 1.00E-124 | 33.47% | 19.19% |
| Nkx2.2(Homeobox)/NPC-Nkx2.2-ChIP-Seq(GSE61673)/Homer | BTBRAGTGSN | 1.00E-122 | 48.94% | 32.77% |
| Twist2(bHLH)/Myoblast-Twist2.Ty1-ChIP-Seq(GSE127998)/Homer | MCAGCTGBYH | 1.00E-122 | 40.76% | 25.45% |
| BMYB(HTH)/Hela-BMYB-ChIP-Seq(GSE27030)/Homer | NHAACBGYYV | 1.00E-121 | 37.13% | 22.43% |
| Bcl6(Zf)/Liver-Bcl6-ChIP-Seq(GSE31578)/Homer | NNNCTTTCCAGGAAA | 1.00E-120 | 32.17% | 18.37% |
| ZNF711(Zf)/SHSY5Y-ZNF711-ChIP-Seq(GSE20673)/Homer | AGGCCTAG | 1.00E-120 | 40.46% | 25.34% |
| NF1-halfsite(CTF)/LNCaP-NF1-ChIP-Seq(Unpublished)/Homer | YTGCCAAG | 1.00E-120 | 40.62% | 25.49% |
| Elf4(ETS)/BMDM-Elf4-ChIP-Seq(GSE88699)/Homer | ACTTCCKGKT | 1.00E-118 | 37.21% | 22.62% |
| MYB(HTH)/ERMYB-Myb-ChIPSeq(GSE22095)/Homer | GGCVGTTR | 1.00E-118 | 39.82% | 24.91% |
| EAR2(NR)/K562-NR2F6-ChIP-Seq(Encode)/Homer | NRBCARRGGTCA | 1.00E-117 | 32.51% | 18.76% |
| Smad2(MAD)/ES-SMAD2-ChIP-Seq(GSE29422)/Homer | CTGTCTGG | 1.00E-117 | 38.00% | 23.36% |
| Znf263(Zf)/K562-Znf263-ChIP-Seq(GSE31477)/Homer | CVGTSCTCCC | 1.00E-117 | 41.26% | 26.22% |
| Hoxd12(Homeobox)/ChickenMSG-Hoxd12.Flag-ChIP-Seq(GSE86088)/Homer | HDGYAATGAAAN | 1.00E-117 | 41.04% | 26.03% |
| COUP-TFII(NR)/Artia-Nr2f2-ChIP-Seq(GSE46497)/Homer | AGRGGTCA | 1.00E-116 | 38.10% | 23.52% |
| LXH9(Homeobox)/Hct116-LXH9.V5-ChIP-Seq(GSE116822)/Homer | NGCTAATTAG | 1.00E-115 | 32.97% | 19.27% |
| Lhx3(Homeobox)/Neuron-Lhx3-ChIP-Seq(GSE31456)/Homer | ADBTAATTAR | 1.00E-114 | 36.09% | 21.91% |
| Rfx6(HTH)/Min6b1-Rfx6.HA-ChIP-Seq(GSE62844)/Homer | TGTTKCCTAGCAACM | 1.00E-114 | 30.03% | 16.95% |
| NeuroG2(bHLH)/Fibroblast-NeuroG2-ChIP-Seq(GSE75910)/Homer | ACCATCTGTT | 1.00E-113 | 37.64% | 23.28% |
| GABPA(ETS)/Jurkat-GABPa-ChIP-Seq(GSE17954)/Homer | RACCGGAAGT | 1.00E-111 | 35.79% | 21.81% |
| Ascl1(bHLH)/NeuralTubes-Ascl1-ChIP-Seq(GSE55840)/Homer | NNVVCAGCTGBN | 1.00E-111 | 39.10% | 24.65% |
| STAT4(Stat)/CD4-Stat4-ChIP-Seq(GSE22104)/Homer | NYTTCCWGGAAR | 1.00E-110 | 27.05% | 14.78% |
| NPAS(bHLH)/Liver-NPAS-ChIP-Seq(GSE39860)/Homer | NVCACGTG | 1.00E-110 | 37.01% | 22.93% |
| KLF14(Zf)/HEK293-KLF14.GFP-ChIP-Seq(GSE58341)/Homer | RGKGGGCGKGGC | 1.00E-109 | 38.56% | 24.30% |
| Olig2(bHLH)/Neuron-Olig2-ChIP-Seq(GSE30882)/Homer | RCCATMTGTT | 1.00E-109 | 41.70% | 27.06% |
| GATA(Zf),IR3/iTreg-Gata3-ChIP-Seq(GSE20898)/Homer | NNNNNBAGATAWYATCTVHN | 1.00E-109 | 10.16% | 3.23% |
| EWS:ERG-fusion(ETS)/CADO_ES1-EWS:ERG-ChIP-Seq(SRA014231)/Homer | ATTTCCTGTN | 1.00E-109 | 31.85% | 18.68% |
| Isl1(Homeobox)/Neuron-Isl1-ChIP-Seq(GSE31456)/Homer | CTAATKGV | 1.00E-108 | 40.08% | 25.71% |
| IRF4(IRF)/GM12878-IRF4-ChIP-Seq(GSE32465)/Homer | ACTGAAACCA | 1.00E-105 | 19.39% | 9.27% |
| ZFX(Zf)/mES-Zfx-ChIP-Seq(GSE11431)/Homer | AGGCCTRG | 1.00E-104 | 33.61% | 20.39% |
| BHLHA15(bHLH)/NIH3T3-BHLHB8.HA-ChIP-Seq(GSE119782)/Homer | NAMCAGCTGK | 1.00E-104 | 34.67% | 21.30% |
| Rbpj1(?)/Panc1-Rbpj1-ChIP-Seq(GSE47459)/Homer | HTTTCCCASG | 1.00E-103 | 32.97% | 19.89% |
| SPDEF(ETS)/VCaP-SPDEF-ChIP-Seq(SRA014231)/Homer | ASWTCCTGBT | 1.00E-103 | 33.97% | 20.74% |
| HIC1(Zf)/Treg-ZBTB29-ChIP-Seq(GSE99889)/Homer | TGCCAGCB | 1.00E-103 | 44.90% | 30.32% |
| HEB(bHLH)/mES-Heb-ChIP-Seq(GSE53233)/Homer | VCAGCTGBNN | 1.00E-102 | 43.96% | 29.55% |
| Tbr1(T-box)/Cortex-Tbr1-ChIP-Seq(GSE71384)/Homer | AAGGTGTKAA | 1.00E-101 | 35.19% | 21.92% |
| GLIS3(Zf)/Thyroid-Glis3.GFP-ChIP-Seq(GSE103297)/Homer | CTCCCTGGGAGGCCN | 1.00E-100 | 40.30% | 26.38% |
| Sox3(HMG)/NPC-Sox3-ChIP-Seq(GSE33059)/Homer | CCWTTGTY | 1.00E-100 | 37.47% | 23.93% |
| Sox6(HMG)/Myotubes-Sox6-ChIP-Seq(GSE32627)/Homer | CCATTGTTNY | 1.00E-99 | 35.17% | 22.03% |
| Tbx21(T-box)/GM12878-TBX21-ChIP-Seq(Encode)/Homer | AGGTGTGAAA | 1.00E-97 | 30.01% | 17.79% |
