# Supplementary material for: Epigenomic characterization of latent HIV infection identifies latency regulating transcription factors
Source: PLoS Pathog. 2021 Feb 26;17(2):e1009346. doi: 10.1371/journal.ppat.1009346 (PMC7946360; doi:10.1371/journal.ppat.1009346)
Supplement: S4 Table — HOMER TF motif enrichment analysis of differentially open peaks between actively infected cells (GFP+) and latently infected (GFP-) cells. Top 100 enriched motifs in peaks preferentially open in latently infected cells are shown. Target Sequences represent genomic sequences that have significantly elevated accessibility in latently infected cells. Background Sequences represent all open chromatin regions in CD4 T cells. (DOC) [file ppat.1009346.s010.doc]

*S4 Table. TF motif enrichment in latently infected cells.*

HOMER TF motif enrichment analysis of differentially open peaks between actively infected cells (GFP+) and latently infected (GFP-) cells. Top 100 enriched motifs in peaks preferentially open in latently infected cells are shown. Target Sequences represent genomic sequences that have significantly elevated accessibility in latently infected cells. Background Sequences represent all open chromatin regions in CD4 T cells.

| Motif Name | Consensus | P-value | % of Target Sequences with Motif | % of Background Sequences with Motif |
| --- | --- | --- | --- | --- |
| FOXP1(Forkhead)/H9-FOXP1-ChIP-Seq(GSE31006)/Homer | NYYTGTTTACHN | 1.00E-32 | 12.02% | 6.79% |
| KLF3(Zf)/MEF-Klf3-ChIP-Seq(GSE44748)/Homer | NRGCCCCRCCCHBNN | 1.00E-32 | 11.11% | 6.13% |
| Foxo3(Forkhead)/U2OS-Foxo3-ChIP-Seq(E-MTAB-2701)/Homer | DGTAAACA | 1.00E-30 | 17.55% | 11.36% |
| Foxa3(Forkhead)/Liver-Foxa3-ChIP-Seq(GSE77670)/Homer | BSNTGTTTACWYWGN | 1.00E-30 | 9.57% | 5.11% |
| ZNF692(Zf)/HEK293-ZNF692.GFP-ChIP-Seq(GSE58341)/Homer | GTGGGCCCCA | 1.00E-29 | 4.87% | 1.94% |
| KLF5(Zf)/LoVo-KLF5-ChIP-Seq(GSE49402)/Homer | DGGGYGKGGC | 1.00E-29 | 19.70% | 13.31% |
| FOXK1(Forkhead)/HEK293-FOXK1-ChIP-Seq(GSE51673)/Homer | NVWTGTTTAC | 1.00E-27 | 21.44% | 14.99% |
| FoxL2(Forkhead)/Ovary-FoxL2-ChIP-Seq(GSE60858)/Homer | WWTRTAAACAVG | 1.00E-27 | 18.89% | 12.86% |
| Foxf1(Forkhead)/Lung-Foxf1-ChIP-Seq(GSE77951)/Homer | WWATRTAAACAN | 1.00E-26 | 19.92% | 13.79% |
| FOXK2(Forkhead)/U2OS-FOXK2-ChIP-Seq(E-MTAB-2204)/Homer | SCHTGTTTACAT | 1.00E-24 | 14.59% | 9.54% |
| Foxa2(Forkhead)/Liver-Foxa2-ChIP-Seq(GSE25694)/Homer | CYTGTTTACWYW | 1.00E-23 | 17.21% | 11.84% |
| KLF6(Zf)/PDAC-KLF6-ChIP-Seq(GSE64557)/Homer | MKGGGYGTGGCC | 1.00E-22 | 16.20% | 11.03% |
| Tbox:Smad(T-box,MAD)/ESCd5-Smad2_3-ChIP-Seq(GSE29422)/Homer | AGGTGHCAGACA | 1.00E-20 | 5.58% | 2.83% |
| Sox9(HMG)/Limb-SOX9-ChIP-Seq(GSE73225)/Homer | AGGVNCCTTTGT | 1.00E-19 | 16.57% | 11.67% |
| Zfp281(Zf)/ES-Zfp281-ChIP-Seq(GSE81042)/Homer | CCCCTCCCCCAC | 1.00E-19 | 4.70% | 2.28% |
| Sox4(HMG)/proB-Sox4-ChIP-Seq(GSE50066)/Homer | YCTTTGTTCC | 1.00E-18 | 15.79% | 11.13% |
| KLF14(Zf)/HEK293-KLF14.GFP-ChIP-Seq(GSE58341)/Homer | RGKGGGCGKGGC | 1.00E-18 | 22.05% | 16.63% |
| FOXA1(Forkhead)/LNCAP-FOXA1-ChIP-Seq(GSE27824)/Homer | WAAGTAAACA | 1.00E-18 | 22.76% | 17.29% |
| Sp2(Zf)/HEK293-Sp2.eGFP-ChIP-Seq(Encode)/Homer | YGGCCCCGCCCC | 1.00E-18 | 19.68% | 14.57% |
| FOXA1(Forkhead)/MCF7-FOXA1-ChIP-Seq(GSE26831)/Homer | WAAGTAAACA | 1.00E-18 | 19.82% | 14.70% |
| FOXM1(Forkhead)/MCF7-FOXM1-ChIP-Seq(GSE72977)/Homer | TRTTTACTTW | 1.00E-17 | 19.97% | 14.90% |
| Klf4(Zf)/mES-Klf4-ChIP-Seq(GSE11431)/Homer | GCCACACCCA | 1.00E-16 | 8.25% | 5.08% |
| EKLF(Zf)/Erythrocyte-Klf1-ChIP-Seq(GSE20478)/Homer | NWGGGTGTGGCY | 1.00E-16 | 5.65% | 3.09% |
| Sp5(Zf)/mES-Sp5.Flag-ChIP-Seq(GSE72989)/Homer | RGKGGGCGGAGC | 1.00E-16 | 14.66% | 10.49% |
| LEF1(HMG)/H1-LEF1-ChIP-Seq(GSE64758)/Homer | CCTTTGATST | 1.00E-14 | 16.01% | 11.81% |
| ZNF7(Zf)/HepG2-ZNF7.Flag-ChIP-Seq(Encode)/Homer | CTGCCWVCTTTTRTA | 1.00E-14 | 12.02% | 8.41% |
| NFAT(RHD)/Jurkat-NFATC1-ChIP-Seq(Jolma_et_al.)/Homer | ATTTTCCATT | 1.00E-13 | 15.88% | 11.93% |
| Sox10(HMG)/SciaticNerve-Sox3-ChIP-Seq(GSE35132)/Homer | CCWTTGTYYB | 1.00E-13 | 25.72% | 20.85% |
| Sox3(HMG)/NPC-Sox3-ChIP-Seq(GSE33059)/Homer | CCWTTGTY | 1.00E-13 | 27.36% | 22.39% |
| Fox:Ebox(Forkhead,bHLH)/Panc1-Foxa2-ChIP-Seq(GSE47459)/Homer | NNNVCTGWGYAAACASN | 1.00E-13 | 18.11% | 13.96% |
| FoxD3(forkhead)/ZebrafishEmbryo-Foxd3.biotin-ChIP-seq(GSE106676)/Homer | TGTTTAYTTAGC | 1.00E-12 | 16.94% | 12.96% |
| TEAD1(TEAD)/HepG2-TEAD1-ChIP-Seq(Encode)/Homer | CYRCATTCCA | 1.00E-12 | 14.76% | 11.06% |
| Sox15(HMG)/CPA-Sox15-ChIP-Seq(GSE62909)/Homer | RAACAATGGN | 1.00E-12 | 18.94% | 14.86% |
| BMYB(HTH)/Hela-BMYB-ChIP-Seq(GSE27030)/Homer | NHAACBGYYV | 1.00E-11 | 23.67% | 19.21% |
| COUP-TFII(NR)/K562-NR2F1-ChIP-Seq(Encode)/Homer | GKBCARAGGTCA | 1.00E-11 | 20.24% | 16.07% |
| EAR2(NR)/K562-NR2F6-ChIP-Seq(Encode)/Homer | NRBCARRGGTCA | 1.00E-11 | 18.31% | 14.33% |
| Foxo1(Forkhead)/RAW-Foxo1-ChIP-Seq(Fan_et_al.)/Homer | CTGTTTAC | 1.00E-11 | 29.81% | 25.06% |
| ZNF341(Zf)/EBV-ZNF341-ChIP-Seq(GSE113194)/Homer | GGAACAGCCG | 1.00E-11 | 11.50% | 8.38% |
| SPDEF(ETS)/VCaP-SPDEF-ChIP-Seq(SRA014231)/Homer | ASWTCCTGBT | 1.00E-11 | 22.66% | 18.43% |
| TEAD3(TEA)/HepG2-TEAD3-ChIP-Seq(Encode)/Homer | TRCATTCCAG | 1.00E-10 | 17.08% | 13.38% |
| Maz(Zf)/HepG2-Maz-ChIP-Seq(GSE31477)/Homer | GGGGGGGG | 1.00E-10 | 15.37% | 11.90% |
| FXR(NR),IR1/Liver-FXR-ChIP-Seq(Chong_et_al.)/Homer | AGGTCANTGACCTB | 1.00E-10 | 7.22% | 4.89% |
| Rfx6(HTH)/Min6b1-Rfx6.HA-ChIP-Seq(GSE62844)/Homer | TGTTKCCTAGCAACM | 1.00E-10 | 16.64% | 13.11% |
| Sox2(HMG)/mES-Sox2-ChIP-Seq(GSE11431)/Homer | BCCATTGTTC | 1.00E-10 | 15.17% | 11.81% |
| TEAD4(TEA)/Tropoblast-Tead4-ChIP-Seq(GSE37350)/Homer | CCWGGAATGY | 1.00E-09 | 12.43% | 9.46% |
| COUP-TFII(NR)/Artia-Nr2f2-ChIP-Seq(GSE46497)/Homer | AGRGGTCA | 1.00E-09 | 22.39% | 18.61% |
| AMYB(HTH)/Testes-AMYB-ChIP-Seq(GSE44588)/Homer | TGGCAGTTGG | 1.00E-09 | 23.01% | 19.18% |
| Smad2(MAD)/ES-SMAD2-ChIP-Seq(GSE29422)/Homer | CTGTCTGG | 1.00E-09 | 21.73% | 18.00% |
| Cdx2(Homeobox)/mES-Cdx2-ChIP-Seq(GSE14586)/Homer | GYMATAAAAH | 1.00E-08 | 13.51% | 10.52% |
| CEBP:AP1(bZIP)/ThioMac-CEBPb-ChIP-Seq(GSE21512)/Homer | DRTGTTGCAA | 1.00E-08 | 15.03% | 11.95% |
| Ets1-distal(ETS)/CD4+-PolII-ChIP-Seq(Barski_et_al.)/Homer | MACAGGAAGT | 1.00E-08 | 12.85% | 10.03% |
| STAT6(Stat)/Macrophage-Stat6-ChIP-Seq(GSE38377)/Homer | TTCCKNAGAA | 1.00E-08 | 10.60% | 8.05% |
| ZFX(Zf)/mES-Zfx-ChIP-Seq(GSE11431)/Homer | AGGCCTRG | 1.00E-08 | 18.31% | 15.03% |
| ZNF467(Zf)/HEK293-ZNF467.GFP-ChIP-Seq(GSE58341)/Homer | TGGGGAAGGGCM | 1.00E-08 | 12.90% | 10.11% |
| Bcl6(Zf)/Liver-Bcl6-ChIP-Seq(GSE31578)/Homer | NNNCTTTCCAGGAAA | 1.00E-07 | 19.73% | 16.40% |
| LRF(Zf)/Erythroblasts-ZBTB7A-ChIP-Seq(GSE74977)/Homer | AAGACCCYYN | 1.00E-07 | 15.83% | 12.82% |
| ZNF189(Zf)/HEK293-ZNF189.GFP-ChIP-Seq(GSE58341)/Homer | TGGAACAGMA | 1.00E-07 | 13.12% | 10.38% |
| KLF10(Zf)/HEK293-KLF10.GFP-ChIP-Seq(GSE58341)/Homer | GGGGGTGTGTCC | 1.00E-07 | 9.47% | 7.14% |
| THRa(NR)/C17.2-THRa-ChIP-Seq(GSE38347)/Homer | GGTCANYTGAGGWCA | 1.00E-07 | 8.10% | 5.95% |
| Zic(Zf)/Cerebellum-ZIC1.2-ChIP-Seq(GSE60731)/Homer | CCTGCTGAGH | 1.00E-07 | 13.14% | 10.42% |
| STAT4(Stat)/CD4-Stat4-ChIP-Seq(GSE22104)/Homer | NYTTCCWGGAAR | 1.00E-07 | 16.62% | 13.59% |
| TEAD(TEA)/Fibroblast-PU.1-ChIP-Seq(Unpublished)/Homer | YCWGGAATGY | 1.00E-07 | 10.28% | 7.87% |
| ETV1(ETS)/GIST48-ETV1-ChIP-Seq(GSE22441)/Homer | AACCGGAAGT | 1.00E-07 | 29.64% | 25.86% |
| Elf4(ETS)/BMDM-Elf4-ChIP-Seq(GSE88699)/Homer | ACTTCCKGKT | 1.00E-07 | 23.79% | 20.32% |
| ZNF711(Zf)/SHSY5Y-ZNF711-ChIP-Seq(GSE20673)/Homer | AGGCCTAG | 1.00E-07 | 21.95% | 18.60% |
| NF1-halfsite(CTF)/LNCaP-NF1-ChIP-Seq(Unpublished)/Homer | YTGCCAAG | 1.00E-07 | 24.67% | 21.23% |
| Stat3+il21(Stat)/CD4-Stat3-ChIP-Seq(GSE19198)/Homer | SVYTTCCNGGAARB | 1.00E-07 | 12.43% | 9.89% |
| Rbpj1(?)/Panc1-Rbpj1-ChIP-Seq(GSE47459)/Homer | HTTTCCCASG | 1.00E-07 | 19.53% | 16.41% |
| Ap4(bHLH)/AML-Tfap4-ChIP-Seq(GSE45738)/Homer | NAHCAGCTGD | 1.00E-06 | 17.62% | 14.67% |
| E2A(bHLH)/proBcell-E2A-ChIP-Seq(GSE21978)/Homer | DNRCAGCTGY | 1.00E-06 | 21.68% | 18.49% |
| Ascl2(bHLH)/ESC-Ascl2-ChIP-Seq(GSE97712)/Homer | SSRGCAGCTGCH | 1.00E-06 | 17.57% | 14.67% |
| Unknown-ESC-element(?)/mES-Nanog-ChIP-Seq(GSE11724)/Homer | CACAGCAGGGGG | 1.00E-06 | 10.65% | 8.35% |
| ERG(ETS)/VCaP-ERG-ChIP-Seq(GSE14097)/Homer | ACAGGAAGTG | 1.00E-06 | 33.55% | 29.87% |
| MITF(bHLH)/MastCells-MITF-ChIP-Seq(GSE48085)/Homer | RTCATGTGAC | 1.00E-06 | 14.71% | 12.05% |
| MyoG(bHLH)/C2C12-MyoG-ChIP-Seq(GSE36024)/Homer | AACAGCTG | 1.00E-06 | 15.42% | 12.71% |
| STAT5(Stat)/mCD4+-Stat5-ChIP-Seq(GSE12346)/Homer | RTTTCTNAGAAA | 1.00E-06 | 7.51% | 5.61% |
| E2A(bHLH),near_PU.1/Bcell-PU.1-ChIP-Seq(GSE21512)/Homer | NVCACCTGBN | 1.00E-06 | 22.08% | 18.97% |
| HNF6(Homeobox)/Liver-Hnf6-ChIP-Seq(ERP000394)/Homer | NTATYGATCH | 1.00E-06 | 9.15% | 7.08% |
| Zac1(Zf)/Neuro2A-Plagl1-ChIP-Seq(GSE75942)/Homer | HAWGRGGCCM | 1.00E-06 | 31.16% | 27.64% |
| Phox2a(Homeobox)/Neuron-Phox2a-ChIP-Seq(GSE31456)/Homer | YTAATYNRATTA | 1.00E-06 | 7.95% | 6.03% |
| bHLHE41(bHLH)/proB-Bhlhe41-ChIP-Seq(GSE93764)/Homer | KCACGTGMCN | 1.00E-06 | 13.58% | 11.10% |
| NFkB-p65(RHD)/GM12787-p65-ChIP-Seq(GSE19485)/Homer | WGGGGATTTCCC | 1.00E-06 | 8.93% | 6.91% |
| TEAD2(TEA)/Py2T-Tead2-ChIP-Seq(GSE55709)/Homer | CCWGGAATGY | 1.00E-06 | 8.03% | 6.11% |
| Klf9(Zf)/GBM-Klf9-ChIP-Seq(GSE62211)/Homer | GCCACRCCCACY | 1.00E-06 | 6.02% | 4.38% |
| Tcf3(HMG)/mES-Tcf3-ChIP-Seq(GSE11724)/Homer | ASWTCAAAGG | 1.00E-06 | 6.73% | 4.99% |
| Etv2(ETS)/ES-ER71-ChIP-Seq(GSE59402)/Homer | NNAYTTCCTGHN | 1.00E-06 | 25.26% | 22.07% |
| ZEB1(Zf)/PDAC-ZEB1-ChIP-Seq(GSE64557)/Homer | VCAGGTRDRY | 1.00E-06 | 25.33% | 22.15% |
| TFE3(bHLH)/MEF-TFE3-ChIP-Seq(GSE75757)/Homer | GTCACGTGACYV | 1.00E-06 | 1.88% | 1.04% |
| VDR(NR),DR3/GM10855-VDR+vitD-ChIP-Seq(GSE22484)/Homer | ARAGGTCANWGAGTTCANNN | 1.00E-06 | 4.04% | 2.73% |
| ZNF264(Zf)/HEK293-ZNF264.GFP-ChIP-Seq(GSE58341)/Homer | RGGGCACTAACY | 1.00E-05 | 10.06% | 7.97% |
| STAT1(Stat)/HelaS3-STAT1-ChIP-Seq(GSE12782)/Homer | NATTTCCNGGAAAT | 1.00E-05 | 6.51% | 4.84% |
| Smad4(MAD)/ESC-SMAD4-ChIP-Seq(GSE29422)/Homer | VBSYGTCTGG | 1.00E-05 | 20.70% | 17.83% |
| USF1(bHLH)/GM12878-Usf1-ChIP-Seq(GSE32465)/Homer | SGTCACGTGR | 1.00E-05 | 7.12% | 5.38% |
| ZNF652/HepG2-ZNF652.Flag-ChIP-Seq(Encode)/Homer | TTAACCCTTTVNKKN | 1.00E-05 | 5.60% | 4.07% |
| EHF(ETS)/LoVo-EHF-ChIP-Seq(GSE49402)/Homer | AVCAGGAAGT | 1.00E-05 | 27.04% | 23.93% |
| Usf2(bHLH)/C2C12-Usf2-ChIP-Seq(GSE36030)/Homer | GTCACGTGGT | 1.00E-05 | 5.90% | 4.36% |
| Tlx?(NR)/NPC-H3K4me1-ChIP-Seq(GSE16256)/Homer | CTGGCAGSCTGCCA | 1.00E-05 | 7.12% | 5.43% |
| Tbx6(T-box)/ESC-Tbx6-ChIP-Seq(GSE93524)/Homer | DAGGTGTBAA | 1.00E-05 | 18.80% | 16.12% |
| c-Jun-CRE(bZIP)/K562-cJun-ChIP-Seq(GSE31477)/Homer | ATGACGTCATCY | 1.00E-05 | 6.17% | 4.60% |
