# Supplementary material for: Epigenomic characterization of latent HIV infection identifies latency regulating transcription factors
Source: PLoS Pathog. 2021 Feb 26;17(2):e1009346. doi: 10.1371/journal.ppat.1009346 (PMC7946360; doi:10.1371/journal.ppat.1009346)
Supplement: S5 Table — The top 50 most highly enriched TF motifs in the set of chromatin peaks that are more open after 24h AZD5582 (250nM) stimulation are shown. Target sequences represent significantly (FDR <0.1) more open chromatin regions after AZD5582 stimulation. Background sequences represent all open chromatin regions in CD4 T cells. (DOC) [file ppat.1009346.s011.doc]

*S5 Table. HOMER motif analysis of AZD5582 stimulated latently infected CD4 T cells*.

The top 50 most highly enriched TF motifs in the set of chromatin peaks that are more open after 24h AZD5582 (250nM) stimulation are shown. Target sequences represent significantly (FDR <0.1) more open chromatin regions after AZD5582 stimulation. Background sequences represent all open chromatin regions in CD4 T cells.

| **Motif Name** | **Motif** | **P-value** | **% of Target Sequences with Motif** | **% of Background Sequences with Motif** |
| --- | --- | --- | --- | --- |
| NFkB-p65(RHD)/GM12787-p65-ChIP-Seq(GSE19485)/Homer | WGGGGATTTCCC | 1e-3254 | 50.12% | 7.12% |
| NFkB-p65-Rel(RHD)/ThioMac-LPS-Expression(GSE23622)/Homer | GGAAATTCCC | 1e-1402 | 13.47% | 0.66% |
| NFkB-p50,p52(RHD)/Monocyte-p50-ChIP-Chip(Schreiber_et_al.)/Homer | GGGGGAATCCCC | 1e-899 | 16.29% | 2.39% |
| Pitx1(Homeobox)/Chicken-Pitx1-ChIP-Seq(GSE38910)/Homer | TAATCCCN | 1e-543 | 68.63% | 45.23% |
| Smad3(MAD)/NPC-Smad3-ChIP-Seq(GSE36673)/Homer | TWGTCTGV | 1e-389 | 55.31% | 35.71% |
| RUNX1(Runt)/Jurkat-RUNX1-ChIP-Seq(GSE29180)/Homer | AAACCACARM | 1e-368 | 38.20% | 21.14% |
| Rbpj1(?)/Panc1-Rbpj1-ChIP-Seq(GSE47459)/Homer | HTTTCCCASG | 1e-366 | 35.99% | 19.40% |
| SCL(bHLH)/HPC7-Scl-ChIP-Seq(GSE13511)/Homer | AVCAGCTG | 1e-358 | 68.96% | 50.07% |
| Nanog(Homeobox)/mES-Nanog-ChIP-Seq(GSE11724)/Homer | RGCCATTAAC | 1e-354 | 63.82% | 44.89% |
| Hoxd11(Homeobox)/ChickenMSG-Hoxd11.Flag-ChIP-Seq(GSE86088)/Homer | VGCCATAAAA | 1e-352 | 47.13% | 29.12% |
| AP-1(bZIP)/ThioMac-PU.1-ChIP-Seq(GSE21512)/Homer | VTGACTCATC | 1e-349 | 27.57% | 13.25% |
| Hoxa13(Homeobox)/ChickenMSG-Hoxa13.Flag-ChIP-Seq(GSE86088)/Homer | CYHATAAAAN | 1e-345 | 48.01% | 30.08% |
| AR-halfsite(NR)/LNCaP-AR-ChIP-Seq(GSE27824)/Homer | CCAGGAACAG | 1e-339 | 61.93% | 43.37% |
| Atf3(bZIP)/GBM-ATF3-ChIP-Seq(GSE33912)/Homer | DATGASTCATHN | 1e-338 | 25.98% | 12.28% |
| BATF(bZIP)/Th17-BATF-ChIP-Seq(GSE39756)/Homer | DATGASTCAT | 1e-335 | 25.62% | 12.05% |
| Tbx5(T-box)/HL1-Tbx5.biotin-ChIP-Seq(GSE21529)/Homer | AGGTGTCA | 1e-335 | 61.63% | 43.20% |
| Eomes(T-box)/H9-Eomes-ChIP-Seq(GSE26097)/Homer | ATTAACACCT | 1e-334 | 46.68% | 29.13% |
| Nkx3.1(Homeobox)/LNCaP-Nkx3.1-ChIP-Seq(GSE28264)/Homer | AAGCACTTAA | 1e-331 | 50.18% | 32.36% |
| Ptf1a(bHLH)/Panc1-Ptf1a-ChIP-Seq(GSE47459)/Homer | ACAGCTGTTN | 1e-330 | 53.22% | 35.21% |
| PRDM10(Zf)/HEK293-PRDM10.eGFP-ChIP-Seq(Encode)/Homer | TGGTACATTCCA | 1e-329 | 21.03% | 8.95% |
| Tgif1(Homeobox)/mES-Tgif1-ChIP-Seq(GSE55404)/Homer | YTGWCADY | 1e-324 | 57.19% | 39.15% |
| Nkx6.1(Homeobox)/Islet-Nkx6.1-ChIP-Seq(GSE40975)/Homer | GKTAATGR | 1e-319 | 46.99% | 29.77% |
| Foxo1(Forkhead)/RAW-Foxo1-ChIP-Seq(Fan_et_al.)/Homer | CTGTTTAC | 1e-319 | 44.25% | 27.36% |
| JunB(bZIP)/DendriticCells-Junb-ChIP-Seq(GSE36099)/Homer | RATGASTCAT | 1e-312 | 23.10% | 10.62% |
| Bcl6(Zf)/Liver-Bcl6-ChIP-Seq(GSE31578)/Homer | NNNCTTTCCAGGAAA | 1e-310 | 32.61% | 17.82% |
| PR(NR)/T47D-PR-ChIP-Seq(GSE31130)/Homer | VAGRACAKNCTGTBC | 1.00E-306 | 45.23% | 28.55% |
| RARa(NR)/K562-RARa-ChIP-Seq(Encode)/Homer | TTGAMCTTTG | 1.00E-306 | 50.78% | 33.58% |
| Hoxa11(Homeobox)/ChickenMSG-Hoxa11.Flag-ChIP-Seq(GSE86088)/Homer | TTTTATGGCM | 1.00E-305 | 45.16% | 28.50% |
| Tgif2(Homeobox)/mES-Tgif2-ChIP-Seq(GSE55404)/Homer | TGTCANYT | 1.00E-305 | 59.47% | 41.90% |
| Isl1(Homeobox)/Neuron-Isl1-ChIP-Seq(GSE31456)/Homer | CTAATKGV | 1.00E-300 | 40.56% | 24.61% |
| Smad4(MAD)/ESC-SMAD4-ChIP-Seq(GSE29422)/Homer | VBSYGTCTGG | 1.00E-300 | 37.76% | 22.25% |
| TRPS1(Zf)/MCF7-TRPS1-ChIP-Seq(GSE107013)/Homer | AGATAAGANN | 1.00E-299 | 43.79% | 27.44% |
| Fra1(bZIP)/BT549-Fra1-ChIP-Seq(GSE46166)/Homer | NNATGASTCATH | 1.00E-298 | 22.81% | 10.64% |
| CRX(Homeobox)/Retina-Crx-ChIP-Seq(GSE20012)/Homer | GCTAATCC | 1.00E-297 | 46.27% | 29.69% |
| Hoxa9(Homeobox)/ChickenMSG-Hoxa9.Flag-ChIP-Seq(GSE86088)/Homer | RGCAATNAAA | 1.00E-297 | 49.06% | 32.23% |
| Twist2(bHLH)/Myoblast-Twist2.Ty1-ChIP-Seq(GSE127998)/Homer | MCAGCTGBYH | 1.00E-294 | 39.53% | 23.88% |
| Nkx2.1(Homeobox)/LungAC-Nkx2.1-ChIP-Seq(GSE43252)/Homer | RSCACTYRAG | 1.00E-293 | 55.38% | 38.25% |
| Hoxd12(Homeobox)/ChickenMSG-Hoxd12.Flag-ChIP-Seq(GSE86088)/Homer | HDGYAATGAAAN | 1.00E-291 | 39.58% | 23.99% |
| EBF2(EBF)/BrownAdipose-EBF2-ChIP-Seq(GSE97114)/Homer | NABTCCCWDGGGAVH | 1.00E-290 | 28.36% | 14.90% |
| Smad2(MAD)/ES-SMAD2-ChIP-Seq(GSE29422)/Homer | CTGTCTGG | 1.00E-288 | 37.12% | 21.98% |
| HEB(bHLH)/mES-Heb-ChIP-Seq(GSE53233)/Homer | VCAGCTGBNN | 1.00E-287 | 43.86% | 27.81% |
| THRb(NR)/Liver-NR1A2-ChIP-Seq(GSE52613)/Homer | TRAGGTCA | 1.00E-287 | 60.37% | 43.32% |
| MYB(HTH)/ERMYB-Myb-ChIPSeq(GSE22095)/Homer | GGCVGTTR | 1.00E-285 | 39.67% | 24.20% |
| BMAL1(bHLH)/Liver-Bmal1-ChIP-Seq(GSE39860)/Homer | GNCACGTG | 1.00E-284 | 38.32% | 23.08% |
| Fra2(bZIP)/Striatum-Fra2-ChIP-Seq(GSE43429)/Homer | GGATGACTCATC | 1.00E-280 | 20.60% | 9.37% |
| NFAT(RHD)/Jurkat-NFATC1-ChIP-Seq(Jolma_et_al.)/Homer | ATTTTCCATT | 1.00E-279 | 24.02% | 11.83% |
| GATA3(Zf)/iTreg-Gata3-ChIP-Seq(GSE20898)/Homer | AGATAASR | 1.00E-278 | 36.84% | 21.97% |
| AMYB(HTH)/Testes-AMYB-ChIP-Seq(GSE44588)/Homer | TGGCAGTTGG | 1.00E-277 | 36.28% | 21.52% |
| Hoxd13(Homeobox)/ChickenMSG-Hoxd13.Flag-ChIP-Seq(GSE86088)/Homer | NCYAATAAAA | 1.00E-276 | 34.69% | 20.25% |
