# Supplementary material for: Epigenomic characterization of latent HIV infection identifies latency regulating transcription factors
Source: PLoS Pathog. 2021 Feb 26;17(2):e1009346. doi: 10.1371/journal.ppat.1009346 (PMC7946360; doi:10.1371/journal.ppat.1009346)
Supplement: S6 Table — The top 50 most highly enriched TF motifs in the set of chromatin peaks that are more open after 24h vorinostat (250nM) stimulation are shown. Target sequences represent significantly (FDR <0.1) more open chromatin regions after vorinostat stimulation. Background sequences represent all open chromatin regions in CD4 T cells. (DOC) [file ppat.1009346.s012.doc]

*S6 Table. HOMER motif analysis of vorinostat stimulated latently infected CD4 T cells*.

The top 50 most highly enriched TF motifs in the set of chromatin peaks that are more open after 24h vorinostat (250nM) stimulation are shown. Target sequences represent significantly (FDR <0.1) more open chromatin regions after vorinostat stimulation. Background sequences represent all open chromatin regions in CD4 T cells.

| **Motif Name** | **Motif** | **P-value** | **% of Target Sequences with Motif** | **% of Background Sequences with Motif** |
| --- | --- | --- | --- | --- |
| Nkx2.1(Homeobox)/LungAC-Nkx2.1-ChIP-Seq(GSE43252)/Homer | RSCACTYRAG | 1.00E-158 | 71.27% | 43.52% |
| Nanog(Homeobox)/mES-Nanog-ChIP-Seq(GSE11724)/Homer | RGCCATTAAC | 1.00E-158 | 75.04% | 47.59% |
| THRb(NR)/Liver-NR1A2-ChIP-Seq(GSE52613)/Homer | TRAGGTCA | 1.00E-156 | 77.08% | 50.03% |
| Smad3(MAD)/NPC-Smad3-ChIP-Seq(GSE36673)/Homer | TWGTCTGV | 1.00E-154 | 69.62% | 42.21% |
| RARa(NR)/K562-RARa-ChIP-Seq(Encode)/Homer | TTGAMCTTTG | 1.00E-151 | 66.15% | 39.00% |
| Znf263(Zf)/K562-Znf263-ChIP-Seq(GSE31477)/Homer | CVGTSCTCCC | 1.00E-146 | 63.93% | 37.31% |
| KLF14(Zf)/HEK293-KLF14.GFP-ChIP-Seq(GSE58341)/Homer | RGKGGGCGKGGC | 1.00E-145 | 66.93% | 40.30% |
| CRX(Homeobox)/Retina-Crx-ChIP-Seq(GSE20012)/Homer | GCTAATCC | 1.00E-144 | 58.85% | 32.72% |
| AR-halfsite(NR)/LNCaP-AR-ChIP-Seq(GSE27824)/Homer | CCAGGAACAG | 1.00E-144 | 75.95% | 49.84% |
| MYB(HTH)/ERMYB-Myb-ChIPSeq(GSE22095)/Homer | GGCVGTTR | 1.00E-143 | 54.47% | 28.92% |
| Tgif2(Homeobox)/mES-Tgif2-ChIP-Seq(GSE55404)/Homer | TGTCANYT | 1.00E-143 | 71.27% | 44.97% |
| KLF5(Zf)/LoVo-KLF5-ChIP-Seq(GSE49402)/Homer | DGGGYGKGGC | 1.00E-142 | 60.33% | 34.25% |
| Tgif1(Homeobox)/mES-Tgif1-ChIP-Seq(GSE55404)/Homer | YTGWCADY | 1.00E-142 | 68.10% | 41.76% |
| Tbx5(T-box)/HL1-Tbx5.biotin-ChIP-Seq(GSE21529)/Homer | AGGTGTCA | 1.00E-141 | 75.13% | 49.23% |
| Eomes(T-box)/H9-Eomes-ChIP-Seq(GSE26097)/Homer | ATTAACACCT | 1.00E-141 | 57.34% | 31.65% |
| Maz(Zf)/HepG2-Maz-ChIP-Seq(GSE31477)/Homer | GGGGGGGG | 1.00E-140 | 60.33% | 34.47% |
| Pitx1(Homeobox)/Chicken-Pitx1-ChIP-Seq(GSE38910)/Homer | TAATCCCN | 1.00E-140 | 74.26% | 48.44% |
| Nkx2.2(Homeobox)/NPC-Nkx2.2-ChIP-Seq(GSE61673)/Homer | BTBRAGTGSN | 1.00E-140 | 61.85% | 35.90% |
| KLF6(Zf)/PDAC-KLF6-ChIP-Seq(GSE64557)/Homer | MKGGGYGTGGCC | 1.00E-139 | 55.77% | 30.40% |
| Rbpj1(?)/Panc1-Rbpj1-ChIP-Seq(GSE47459)/Homer | HTTTCCCASG | 1.00E-139 | 49.09% | 24.73% |
| AMYB(HTH)/Testes-AMYB-ChIP-Seq(GSE44588)/Homer | TGGCAGTTGG | 1.00E-138 | 50.04% | 25.54% |
| Erra(NR)/HepG2-Erra-ChIP-Seq(GSE31477)/Homer | CAAAGGTCAG | 1.00E-138 | 61.81% | 36.03% |
| Zac1(Zf)/Neuro2A-Plagl1-ChIP-Seq(GSE75942)/Homer | HAWGRGGCCM | 1.00E-137 | 72.53% | 46.83% |
| ZNF416(Zf)/HEK293-ZNF416.GFP-ChIP-Seq(GSE58341)/Homer | WDNCTGGGCA | 1.00E-137 | 52.73% | 27.93% |
| Bcl6(Zf)/Liver-Bcl6-ChIP-Seq(GSE31578)/Homer | NNNCTTTCCAGGAAA | 1.00E-137 | 45.40% | 21.90% |
| COUP-TFII(NR)/Artia-Nr2f2-ChIP-Seq(GSE46497)/Homer | AGRGGTCA | 1.00E-136 | 54.25% | 29.35% |
| Hoxd11(Homeobox)/ChickenMSG-Hoxd11.Flag-ChIP-Seq(GSE86088)/Homer | VGCCATAAAA | 1.00E-135 | 55.25% | 30.29% |
| Hoxa13(Homeobox)/ChickenMSG-Hoxa13.Flag-ChIP-Seq(GSE86088)/Homer | CYHATAAAAN | 1.00E-135 | 55.30% | 30.33% |
| Nkx2.5(Homeobox)/HL1-Nkx2.5.biotin-ChIP-Seq(GSE21529)/Homer | RRSCACTYAA | 1.00E-135 | 62.24% | 36.71% |
| HIC1(Zf)/Treg-ZBTB29-ChIP-Seq(GSE99889)/Homer | TGCCAGCB | 1.00E-134 | 64.11% | 38.52% |
| SCL(bHLH)/HPC7-Scl-ChIP-Seq(GSE13511)/Homer | AVCAGCTG | 1.00E-134 | 80.82% | 56.42% |
| BMYB(HTH)/Hela-BMYB-ChIP-Seq(GSE27030)/Homer | NHAACBGYYV | 1.00E-134 | 49.18% | 25.18% |
| Nkx3.1(Homeobox)/LNCaP-Nkx3.1-ChIP-Seq(GSE28264)/Homer | AAGCACTTAA | 1.00E-134 | 61.98% | 36.55% |
| Sp2(Zf)/HEK293-Sp2.eGFP-ChIP-Seq(Encode)/Homer | YGGCCCCGCCCC | 1.00E-133 | 62.89% | 37.47% |
| Olig2(bHLH)/Neuron-Olig2-ChIP-Seq(GSE30882)/Homer | RCCATMTGTT | 1.00E-133 | 54.56% | 29.86% |
| Sp5(Zf)/mES-Sp5.Flag-ChIP-Seq(GSE72989)/Homer | RGKGGGCGGAGC | 1.00E-132 | 56.03% | 31.20% |
| NF1-halfsite(CTF)/LNCaP-NF1-ChIP-Seq(Unpublished)/Homer | YTGCCAAG | 1.00E-132 | 55.77% | 30.97% |
| Smad2(MAD)/ES-SMAD2-ChIP-Seq(GSE29422)/Homer | CTGTCTGG | 1.00E-131 | 53.60% | 29.16% |
| Hoxa9(Homeobox)/ChickenMSG-Hoxa9.Flag-ChIP-Seq(GSE86088)/Homer | RGCAATNAAA | 1.00E-130 | 58.16% | 33.35% |
| Ptf1a(bHLH)/Panc1-Ptf1a-ChIP-Seq(GSE47459)/Homer | ACAGCTGTTN | 1.00E-130 | 68.75% | 43.61% |
| LRF(Zf)/Erythroblasts-ZBTB7A-ChIP-Seq(GSE74977)/Homer | AAGACCCYYN | 1.00E-128 | 56.77% | 32.24% |
| Smad4(MAD)/ESC-SMAD4-ChIP-Seq(GSE29422)/Homer | VBSYGTCTGG | 1.00E-128 | 53.86% | 29.67% |
| KLF10(Zf)/HEK293-KLF10.GFP-ChIP-Seq(GSE58341)/Homer | GGGGGTGTGTCC | 1.00E-128 | 38.45% | 17.22% |
| Twist2(bHLH)/Myoblast-Twist2.Ty1-ChIP-Seq(GSE127998)/Homer | MCAGCTGBYH | 1.00E-127 | 53.82% | 29.69% |
| Bapx1(Homeobox)/VertebralCol-Bapx1-ChIP-Seq(GSE36672)/Homer | TTRAGTGSYK | 1.00E-127 | 60.98% | 36.26% |
| COUP-TFII(NR)/K562-NR2F1-ChIP-Seq(Encode)/Homer | GKBCARAGGTCA | 1.00E-126 | 48.61% | 25.33% |
| Meis1(Homeobox)/MastCells-Meis1-ChIP-Seq(GSE48085)/Homer | VGCTGWCAVB | 1.00E-124 | 53.95% | 30.07% |
| GLIS3(Zf)/Thyroid-Glis3.GFP-ChIP-Seq(GSE103297)/Homer | CTCCCTGGGAGGCCN | 1.00E-123 | 62.07% | 37.59% |
| TRPS1(Zf)/MCF7-TRPS1-ChIP-Seq(GSE107013)/Homer | AGATAAGANN | 1.00E-123 | 50.87% | 27.47% |
