# Supplementary material for: Epigenomic characterization of latent HIV infection identifies latency regulating transcription factors
Source: PLoS Pathog. 2021 Feb 26;17(2):e1009346. doi: 10.1371/journal.ppat.1009346 (PMC7946360; doi:10.1371/journal.ppat.1009346)
Supplement: S7 Table — The top 50 most highly enriched TF motifs in the set of chromatin peaks that are more open after 24h prostratin stimulation (250nM) are shown. Target sequences represent significantly (FDR <0.1) more open chromatin regions after prostratin stimulation. Background sequences represent all open chromatin regions in CD4 T cells. (DOCX) [file ppat.1009346.s013.docx]

*S7 Table. HOMER motif analysis of prostratin stimulated latently infected CD4 T cells*.

The top 50 most highly enriched TF motifs in the set of chromatin peaks that are more open after 24h prostratin stimulation (250nM) are shown. Target sequences represent significantly (FDR <0.1) more open chromatin regions after prostratin stimulation. Background sequences represent all open chromatin regions in CD4 T cells.

| **Motif Name** | **Motif** | **P-value** | **% of Target Sequences with Motif** | **% of Background Sequences with Motif** |
| --- | --- | --- | --- | --- |
| Fra1(bZIP)/BT549-Fra1-ChIP-Seq(GSE46166)/Homer | NNATGASTCATH | 1e-8945 | 46.16% | 6.17% |
| Atf3(bZIP)/GBM-ATF3-ChIP-Seq(GSE33912)/Homer | DATGASTCATHN | 1e-8905 | 49.80% | 7.65% |
| BATF(bZIP)/Th17-BATF-ChIP-Seq(GSE39756)/Homer | DATGASTCAT | 1e-8844 | 49.27% | 7.50% |
| JunB(bZIP)/DendriticCells-Junb-ChIP-Seq(GSE36099)/Homer | RATGASTCAT | 1e-8731 | 45.82% | 6.26% |
| Fra2(bZIP)/Striatum-Fra2-ChIP-Seq(GSE43429)/Homer | GGATGACTCATC | 1e-8418 | 42.05% | 5.22% |
| AP-1(bZIP)/ThioMac-PU.1-ChIP-Seq(GSE21512)/Homer | VTGACTCATC | 1e-8322 | 50.28% | 8.59% |
| Fosl2(bZIP)/3T3L1-Fosl2-ChIP-Seq(GSE56872)/Homer | NATGASTCABNN | 1e-7274 | 33.76% | 3.54% |
| Jun-AP1(bZIP)/K562-cJun-ChIP-Seq(GSE31477)/Homer | GATGASTCATCN | 1e-6346 | 27.54% | 2.49% |
| Bach2(bZIP)/OCILy7-Bach2-ChIP-Seq(GSE44420)/Homer | TGCTGAGTCA | 1e-2687 | 16.00% | 2.21% |
| Nanog(Homeobox)/mES-Nanog-ChIP-Seq(GSE11724)/Homer | RGCCATTAAC | 1e-1043 | 56.62% | 37.70% |
| Pitx1(Homeobox)/Chicken-Pitx1-ChIP-Seq(GSE38910)/Homer | TAATCCCN | 1e-970 | 54.95% | 36.75% |
| MafK(bZIP)/C2C12-MafK-ChIP-Seq(GSE36030)/Homer | GCTGASTCAGCA | 1e-963 | 9.75% | 2.35% |
| MafA(bZIP)/Islet-MafA-ChIP-Seq(GSE30298)/Homer | TGCTGACTCA | 1e-955 | 20.40% | 8.53% |
| RUNX1(Runt)/Jurkat-RUNX1-ChIP-Seq(GSE29180)/Homer | AAACCACARM | 1e-908 | 30.49% | 16.11% |
| SCL(bHLH)/HPC7-Scl-ChIP-Seq(GSE13511)/Homer | AVCAGCTG | 1e-900 | 58.73% | 41.03% |
| Tbx5(T-box)/HL1-Tbx5.biotin-ChIP-Seq(GSE21529)/Homer | AGGTGTCA | 1e-890 | 54.00% | 36.58% |
| CRX(Homeobox)/Retina-Crx-ChIP-Seq(GSE20012)/Homer | GCTAATCC | 1e-873 | 38.45% | 22.82% |
| Nkx6.1(Homeobox)/Islet-Nkx6.1-ChIP-Seq(GSE40975)/Homer | GKTAATGR | 1e-867 | 40.94% | 24.98% |
| Hoxa13(Homeobox)/ChickenMSG-Hoxa13.Flag-ChIP-Seq(GSE86088)/Homer | CYHATAAAAN | 1e-846 | 39.58% | 23.99% |
| Tgif1(Homeobox)/mES-Tgif1-ChIP-Seq(GSE55404)/Homer | YTGWCADY | 1e-843 | 52.23% | 35.36% |
| Hoxd11(Homeobox)/ChickenMSG-Hoxd11.Flag-ChIP-Seq(GSE86088)/Homer | VGCCATAAAA | 1e-833 | 38.43% | 23.12% |
| RUNX2(Runt)/PCa-RUNX2-ChIP-Seq(GSE33889)/Homer | NWAACCACADNN | 1e-829 | 26.33% | 13.44% |
| TRPS1(Zf)/MCF7-TRPS1-ChIP-Seq(GSE107013)/Homer | AGATAAGANN | 1e-826 | 37.45% | 22.35% |
| Eomes(T-box)/H9-Eomes-ChIP-Seq(GSE26097)/Homer | ATTAACACCT | 1e-821 | 38.81% | 23.54% |
| Pdx1(Homeobox)/Islet-Pdx1-ChIP-Seq(SRA008281)/Homer | YCATYAATCA | 1e-820 | 21.13% | 9.71% |
| Bapx1(Homeobox)/VertebralCol-Bapx1-ChIP-Seq(GSE36672)/Homer | TTRAGTGSYK | 1e-817 | 41.85% | 26.20% |
| Nkx3.1(Homeobox)/LNCaP-Nkx3.1-ChIP-Seq(GSE28264)/Homer | AAGCACTTAA | 1e-808 | 41.91% | 26.33% |
| Tgif2(Homeobox)/mES-Tgif2-ChIP-Seq(GSE55404)/Homer | TGTCANYT | 1e-806 | 54.21% | 37.59% |
| Smad3(MAD)/NPC-Smad3-ChIP-Seq(GSE36673)/Homer | TWGTCTGV | 1e-802 | 43.81% | 28.06% |
| Foxo1(Forkhead)/RAW-Foxo1-ChIP-Seq(Fan_et_al.)/Homer | CTGTTTAC | 1e-797 | 34.76% | 20.34% |
| Hoxa9(Homeobox)/ChickenMSG-Hoxa9.Flag-ChIP-Seq(GSE86088)/Homer | RGCAATNAAA | 1e-786 | 41.67% | 26.31% |
| Hoxa11(Homeobox)/ChickenMSG-Hoxa11.Flag-ChIP-Seq(GSE86088)/Homer | TTTTATGGCM | 1e-783 | 37.57% | 22.81% |
| AR-halfsite(NR)/LNCaP-AR-ChIP-Seq(GSE27824)/Homer | CCAGGAACAG | 1e-778 | 50.37% | 34.25% |
| BMAL1(bHLH)/Liver-Bmal1-ChIP-Seq(GSE39860)/Homer | GNCACGTG | 1e-762 | 32.55% | 18.79% |
| Nkx2.1(Homeobox)/LungAC-Nkx2.1-ChIP-Seq(GSE43252)/Homer | RSCACTYRAG | 1e-760 | 47.50% | 31.78% |
| Nkx2.5(Homeobox)/HL1-Nkx2.5.biotin-ChIP-Seq(GSE21529)/Homer | RRSCACTYAA | 1e-757 | 40.54% | 25.58% |
| GATA3(Zf)/iTreg-Gata3-ChIP-Seq(GSE20898)/Homer | AGATAASR | 1e-754 | 30.80% | 17.44% |
| NF-E2(bZIP)/K562-NFE2-ChIP-Seq(GSE31477)/Homer | GATGACTCAGCA | 1e-748 | 4.86% | 0.72% |
| Tbr1(T-box)/Cortex-Tbr1-ChIP-Seq(GSE71384)/Homer | AAGGTGTKAA | 1e-723 | 28.01% | 15.46% |
| Hoxd12(Homeobox)/ChickenMSG-Hoxd12.Flag-ChIP-Seq(GSE86088)/Homer | HDGYAATGAAAN | 1e-722 | 32.77% | 19.29% |
| Isl1(Homeobox)/Neuron-Isl1-ChIP-Seq(GSE31456)/Homer | CTAATKGV | 1e-704 | 32.31% | 19.07% |
| Egr1(Zf)/K562-Egr1-ChIP-Seq(GSE32465)/Homer | TGCGTGGGYG | 1e-703 | 16.88% | 7.40% |
| RARa(NR)/K562-RARa-ChIP-Seq(Encode)/Homer | TTGAMCTTTG | 1e-703 | 40.35% | 25.92% |
| Ptf1a(bHLH)/Panc1-Ptf1a-ChIP-Seq(GSE47459)/Homer | ACAGCTGTTN | 1e-702 | 41.28% | 26.74% |
| NPAS(bHLH)/Liver-NPAS-ChIP-Seq(GSE39860)/Homer | NVCACGTG | 1e-696 | 28.97% | 16.42% |
| Bach1(bZIP)/K562-Bach1-ChIP-Seq(GSE31477)/Homer | AWWNTGCTGAGTCAT | 1e-690 | 4.60% | 0.70% |
| MafB(bZIP)/BMM-Mafb-ChIP-Seq(GSE75722)/Homer | WNTGCTGASTCAGCANWTTY | 1e-689 | 12.07% | 4.39% |
| Tbet(T-box)/CD8-Tbet-ChIP-Seq(GSE33802)/Homer | AGGTGTGAAM | 1e-686 | 24.82% | 13.24% |
| Sox10(HMG)/SciaticNerve-Sox3-ChIP-Seq(GSE35132)/Homer | CCWTTGTYYB | 1e-686 | 28.19% | 15.88% |
