# Supplementary material for: Epigenomic characterization of latent HIV infection identifies latency regulating transcription factors
Source: PLoS Pathog. 2021 Feb 26;17(2):e1009346. doi: 10.1371/journal.ppat.1009346 (PMC7946360; doi:10.1371/journal.ppat.1009346)
Supplement: S8 Table — Change in the mean of the ChIPseq TF score for significantly more accessible peaks (FC > 1.5 and FDR < 0.1, sig) vs non-significantly different (unchanged) peaks (FDR > 0.2 and any FC, nonSig). Calculated using Welch’s Two Sample t-test. Box plots of distributions are shown in S5 Fig. AZD = AZD5582, PROST = prostratin, VOR = vorinostat. (DOCX) [file ppat.1009346.s014.docx]

*S8 Table. Enrichment of ATACseq peaks for ChIPseq validated transcription factor binding sites.*

Change in the mean of the ChIPseq TF score for significantly more accessible peaks (FC > 1.5 and FDR < 0.1, sig) vs non-significantly different (unchanged) peaks (FDR > 0.2 and any FC, nonSig). Calculated using Welch’s Two Sample t-test. Box plots of distributions are shown in S5 Fig. AZD=AZD5582, PROST=prostratin, VOR=vorinostat.

| **Data** | **TF** | **p-Value** | **Sig. Count** | **Non-Sig.**  **Count** | **Mean Sig.** | **Mean Diff.** | **95% Conf Diff. Low** | **95% Conf Diff. High** |
| --- | --- | --- | --- | --- | --- | --- | --- | --- |
| GFP+ vs GFP- | RUNX1 | 6.18E-168 | 3773 | 271408 | 2.2341 | 0.9381 | 0.8747 | 1.0014 |
| GFP+ vs GFP- | GATA3 | 5.76E-236 | 3773 | 271408 | 2.0501 | 0.8831 | 0.8340 | 0.9322 |
| GFP+ vs GFP- | MYB | 1.30E-124 | 3773 | 271408 | 1.7863 | 0.5807 | 0.5345 | 0.6269 |
| GFP+ vs GFP- | CTCF | 1.37E-28 | 3773 | 271408 | 1.4983 | 0.2382 | 0.1965 | 0.2800 |
|  |  |  |  |  |  |  |  |  |
| AZD | RUNX1 | 3.78E-19 | 8177 | 146312 | 1.7763 | 0.1921 | 0.1501 | 0.2341 |
| AZD | GATA3 | 4.89E-30 | 8177 | 146312 | 1.5514 | 0.1727 | 0.1431 | 0.2023 |
| AZD | MYB | 3.45E-13 | 8177 | 146312 | 1.5620 | 0.1195 | 0.0874 | 0.1517 |
| PROST | RUNX1 | 3.80E-01 | 27102 | 99106 | 1.3704 | 0.0097 | -0.0120 | 0.0314 |
| PROSTR | GATA3 | 6.59E-44 | 27102 | 99106 | 1.3036 | 0.1111 | 0.0954 | 0.1267 |
| VOR | MYB | 2.93E-01 | 782 | 146391 | 1.6166 | 0.0521 | -0.0451 | 0.1494 |
